# MoGe2-SLAM: Outdoor Monocular 3D Gaussian Splatting SLAM with Absolute Scale

**DOI:** 10.3390/s26206388

**Published:** 2026-10-09

**Authors:** Jiaen Zhao, Jinming Zhang, Biqing Li, Haitao Luo, Mengyu Zhu, Xiongfei Liu, Lili Zhang

**Affiliations:** 1 Aerospace Information Research Institute, Chinese Academy of Sciences, Beijing 100190, China; zhaojiaen24@mails.ucas.ac.cn (J.Z.); nicnyzjm@whu.edu.cn (J.Z.); libiqing22@mails.ucas.ac.cn (B.L.); luohaitao21@mails.ucas.ac.cn (H.L.); zhumengyu25@mails.ucas.ac.cn (M.Z.); liuxf004327@aircas.ac.cn (X.L.); 2 Key Laboratory of Target Cognition and Application Technology (TCAT), Aerospace Information Research Institute, Chinese Academy of Sciences, Beijing 100190, China; 3 University of Chinese Academy of Sciences, Beijing 100049, China; 4 School of Electronic, Electrical and Communication Engineering, University of Chinese Academy of Sciences, Beijing 100049, China

**Keywords:** visual SLAM, 3D Gaussian splatting, absolute-scale estimation, outdoor scene reconstruction

## Abstract

3D Gaussian Splatting (3DGS) has significantly advanced the field of visual SLAM. However, outdoor monocular SLAM systems still suffer from scale ambiguity, so recovering the absolute scale remains difficult, and tracking often becomes brittle in large scenes. To address these issues, we present MoGe2-SLAM, an outdoor monocular 3DGS-SLAM framework that incrementally recovers camera trajectories and dense maps in an approximate absolute metric scale from a monocular RGB sequence with a known camera-height prior. By approximate absolute metric scale, we mean that camera poses and the reconstructed map are expressed in meters after camera-height calibration, rather than only up to an unknown global scale. Our core idea is to anchor frozen MoGe-2 geometry to a physical camera-height/ground-plane ruler, enabling approximate absolute-scale reconstruction. Specifically, we perform Absolute-Scale Initialization on the first keyframe by replacing MoGe-2’s advertised metric global scale with a known camera height and ground-plane geometry, generating the initial Gaussian scene directly in physical units. We then adopt Grid-Balanced Tracking that encourages spatially distributed feature extraction and maintains stable tracking. For mapping, we employ an ORB-anchored strategy that aligns the MoGe-2 depth prior with the rendered depth using geometric constraints, thereby mitigating scale drift. On KITTI, Waymo, and Argoverse 2, MoGe2-SLAM is competitive with existing monocular SLAM baselines in trajectory shape and novel-view fidelity while recovering trajectories and Gaussian maps in an approximate physical scale under the stated camera-height prior.

## 1. Introduction

**Figure 1:**
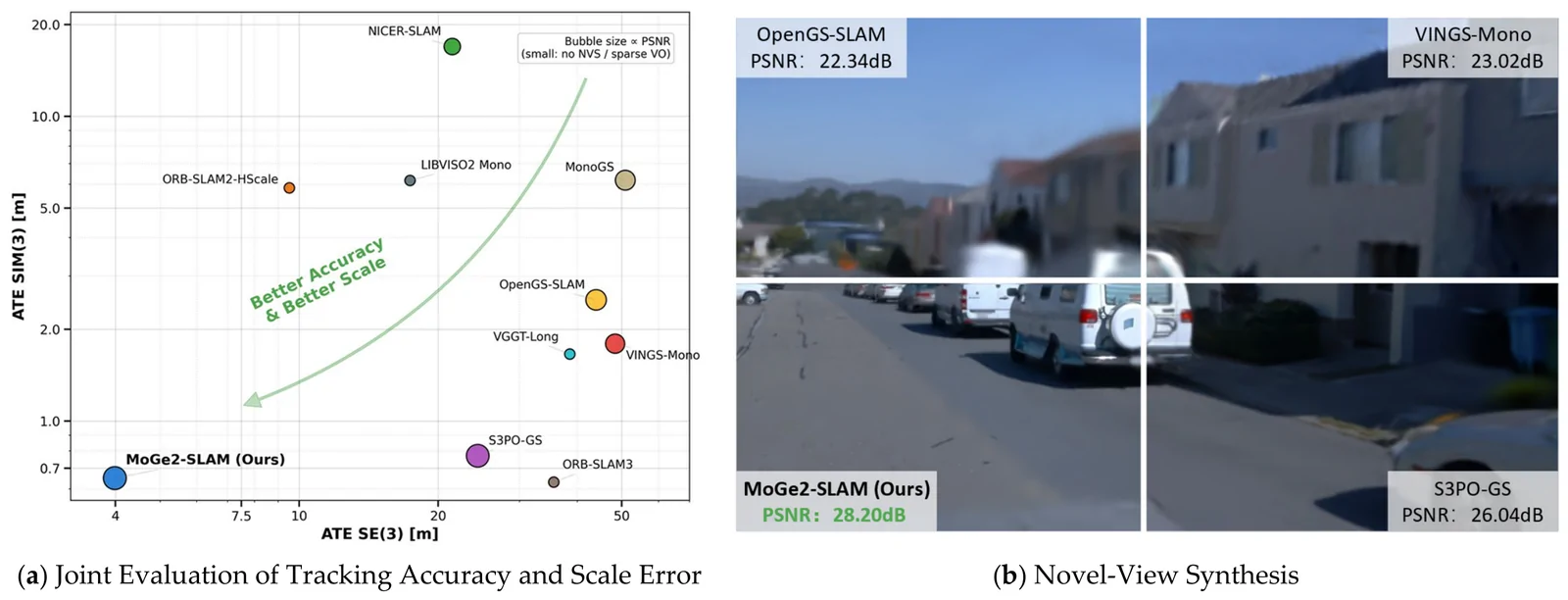
Performance overview of MoGe2-SLAM. (**a**) Joint evaluation of absolute-scale tracking accuracy (ATE-SE(3), x-axis) and trajectory-shape fidelity under Sim(3) alignment (ATE-Sim(3), y-axis); bubble size is proportional to novel-view PSNR. MoGe2-SLAM resides in the bottom-left corner, demonstrating superior tracking accuracy and approximate absolute metric scale recovery compared with state-of-the-art methods such as S3PO-GS, VINGS-Mono, and OpenGS-SLAM. (**b**) Qualitative comparison of novel-view synthesis. MoGe2-SLAM reconstructs high-fidelity outdoor details with competitive PSNR, consistent with baseline approaches.

Simultaneous Localization and Mapping (SLAM) [1] serves as a cornerstone technology for intelligent systems, encompassing autonomous driving, mobile robotics, and virtual reality. With the emergence of 3D Gaussian Splatting (3DGS) [2], this domain has undergone a paradigm shift: SLAM outputs are evolving from sparse feature point clouds toward photorealistic, pixel-level scene representations. Leveraging the explicit scene representation and efficient differentiable rendering of 3DGS, the visual quality of maps is significantly enhanced, thereby improving their utility for downstream tasks. Our MoGe2 SLAM is a 3DGS-SLAM system. We have summarized the comparison between MoGe2 SLAM and outdoor monocular 3DGS baseline methods in terms of tracking rendering trade-offs, as shown in Figure 1.

Current research on monocular 3DGS SLAM predominantly targets indoor environments, where its remarkable success stems from three key factors. First, indoor scenes benefit from rich structural priors, often adhering to the Manhattan World assumption [3], where regular geometric structures facilitate indirect scale estimation. Second, the restricted motion space limits exploration ranges, enabling frequent loop closures that support multi-view optimization, such as bundle adjustment [4], to effectively maintain local scale consistency. Third, controllable lighting conditions provide stable diffuse environments, which not only promote robust feature tracking but also ensure the reliability of photometric optimization.

Monocular 3DGS SLAM faces severe challenges in large-scale outdoor environments, where scale ambiguity is the core bottleneck [5,6]. The openness of outdoor scenes exacerbates depth uncertainty: without known geometric references, the system struggles to converge to the correct metric scale. Outdoor imagery is further dominated by weakly textured regions (sky and road surfaces) and by unstructured, repetitively textured objects such as vegetation. These factors disrupt feature matching and degrade depth prediction, ultimately causing substantial scale drift or tracking failure [7].

Integrating deep learning into SLAM has become a dominant research trend [8,9,10]. Early networks trained on limited indoor data generalize poorly outdoors, whereas foundation models pretrained at the web scale [11,12,13] offer stronger geometric priors. Even so, most predictors still return relative depth; injecting them unchanged into high-precision SLAM often warps the structure and fails to yield maps that are usable for metric perception.

Consequently, the scientific bottleneck is no longer depth acquisition alone but the coupling between strong data-driven priors and geometrically rigorous SLAM. We present MoGe2-SLAM, a metric monocular 3DGS SLAM system for outdoor driving under single-camera RGB sensing with an explicit camera-height and ground-plane prior. The approach metrically anchors a frozen MoGe-2 [13] depth prior through Absolute-Scale Initialization, combines Grid-Balanced Tracking in the front end with ORB-Anchored Mapping in the back end, and propagates a consistent metric scale online without auxiliary ranging or inertial sensors.

Our main contributions are as follows:

1. We propose a monocular 3DGS-based visual SLAM system that recovers the absolute physical scale in outdoor scenarios using only a single RGB stream and known camera height.
2. We develop a geometry-aware Absolute-Scale Initialization strategy that metrically rescales and refines precomputed depth maps prior to Gaussian initialization, establishing a physically grounded geometric basis for metric map reconstruction.
3. We present a coupled framework of Grid-Balanced Tracking and ORB-Anchored Mapping. By exploiting spatial feature uniformity and sparse keypoint alignment principles, the front end and back end collaboratively constrain cross-frame geometric consistency and enhance long-term scale stability.

The remainder of this paper is organized as follows: Section 2 reviews related work. Section 3 presents the problem formulation and method of MoGe2-SLAM. Section 4 reports experiments on KITTI, Waymo, and Argoverse 2. Section 5 discusses limitations, and Section 6 concludes the paper.

## 2. Related Work

### 2.1. Classical Monocular SLAM

Classical monocular SLAM achieves real-time localization from a single camera and is commonly grouped by data association [6,14]. Feature-based systems estimate motion from salient keypoints. MonoSLAM [15] tackled the then-open problem of real-time monocular filtering with an EKF that jointly estimates camera pose and a sparse landmark map. PTAM [16] abandoned the filter in favor of parallel tracking and mapping threads with nonlinear back-end optimization, trading per-frame latency for global accuracy and enabling interactive AR on small workspaces. The ORB-SLAM family [17,18,19] then set the standard for long-term robustness: ORB-SLAM [17] introduced full loop closure, ORB-SLAM2 [18] extended the design to stereo and RGB-D, and ORB-SLAM3 [19] added multi-map and visual–inertial support, establishing a widely used accuracy–robustness benchmark.

Direct methods instead minimize photometric errors to cope with texture-poor or high-frame-rate sensing. DTAM [20] pioneered dense reconstruction from monocular video with a Graphics Processing Unit (GPU) photometric volume pipeline, at a substantial computational cost. LSD-SLAM [21] scaled semi-dense mapping beyond small rooms by tracking high-gradient pixels on a keyframe-based direct back end. Direct sparse odometry formulations [22,23] jointly optimize pose, inverse depth, and (when needed) affine brightness, yielding strong visual odometry without descriptors. Semi-direct designs such as SVO [24] track patches directly while maintaining a feature-based map, striking an accuracy–efficiency trade-off for compute-constrained platforms.

Despite accurate localization, these pipelines typically output sparse or semi-dense point clouds that cannot recover continuous surface appearance. More critically for outdoor metric perception, triangulation from monocular motion remains theoretically scale-ambiguous: without an external ruler, the absolute metric scale is unobservable, and long non-looping drives offer few opportunities for classical loop-closure correction of accumulated drift [4,25]. Offline multi-view reconstruction such as COLMAP [26] can refine geometry when wide baselines and sufficient overlaps exist, yet it does not provide an online absolute ruler for forward-facing monocular driving.

### 2.2. Neural Implicit Mapping and 3DGS-SLAM

Neural Radiance Fields (NeRF) [27] reshaped high-fidelity mapping by representing scenes as continuous radiance fields. iMAP [28] brought this representation into online dense SLAM, jointly optimizing the camera pose and a compact MLP in an early RGB-D NeRF-SLAM. Follow-up implicit systems then attacked the scalability of monolithic MLPs: NICE-SLAM [29] replaced them with hierarchical grid encodings that support larger rooms, Vox-Fusion [30] added incremental expansion into unknown space through voxel-based dynamic allocation, Co-SLAM [31] introduced joint coordinate and parametric encodings that balance memory and speed for real-time neural SLAM, and Point-SLAM [32] moved to a dense neural point-cloud formulation. NeRF-SLAM [33] and NICER-SLAM [34] extended these designs to dense monocular or RGB input, tightening the coupling between tracking and neural scene encoding. These systems deliver continuous geometry and appearance, yet implicit optimization is often too slow or memory-heavy for long outdoor sequences, and RGB-D variants still rely on metric depth hardware.

3D Gaussian Splatting [2] offers an explicit, real-time alternative that targets photorealistic rendering with differentiable rasterization. SplaTAM [35] first demonstrated dense 3DGS SLAM on RGB-D input, consuming measured depth to resolve monocular scale ambiguity within a splat–track–map pipeline. MonoGS [36] then removed the depth sensor: its evidence that differentiable rendering alone can drive online pose and map updates made RGB-only 3DGS-SLAM viable, though the recovered geometry remains Sim(3)-ambiguous. Subsequent systems such as Photo-SLAM [37], GS-SLAM [38], MonoGS++ [39], and Gaussian-SLAM [40] refined photorealistic mapping, primitive control, or initialization speed across camera modalities. Outdoor systems then diversified around metric recovery and unbounded scenes. VINGS-Mono [41] sought metric monocular operation with a tightly coupled VIO front end, recovering the absolute scale from inertial constraints when an IMU is available. OpenGS-SLAM [42] tackled unbounded outdoor mapping by combining point-map regression with differentiable Gaussian optimization. S3PO-GS [43] built outdoor monocular 3DGS on foundation-model geometry, injecting MASt3R-based [44] priors for coarse scale cues, although cross-domain generalization of such priors remains delicate. Collectively, 3DGS-SLAM achieves competitive novel-view quality, but purely monocular variants remain scale-ambiguous, photometric-heavy pipelines remain brittle on weak outdoor texture, and sensor-augmented or prior-only designs still leave absolute, online metric consistency unresolved under single-camera sensing.

### 2.3. Absolute-Scale Recovery Under Single-Camera Sensing

The absolute scale connects reconstructed geometry to physical use. Existing remedies fall into several families. Geometric-prior/ground-plane visual odometry (VO) methods [45,46,47,48], including classic height-over-ground monocular VO such as LIBVISO2 Mono [45], recover a physical ruler from known object sizes or a dominant road plane with a known camera mounting height; Zhou-style ground-plane absolute-scale estimation [46] and its open ORB-SLAM2-HScale [46] reimplementation are representative of this classical metric-VO line. They provide sensor-free metric cues under driving assumptions yet typically rescale a sparse VO back end episodically rather than maintaining a dense photorealistic map. Multi-sensor fusion, especially IMU-based VIO [49,50,51,52] and visual-inertial 3DGS systems such as VINGS-Mono [41], recovers the gravity-aligned metric scale when an IMU is available, at the cost of calibration complexity and a departure from single-camera sensing. RGB-D/measured-depth Gaussian SLAM (e.g., SplaTAM [35]) resolves the scale by consuming a metric depth stream, which is outside our RGB-only outdoor setting. Back-end loop closure and global bundle adjustment [4,25] reduce long-horizon drift after revisits, but they cannot create an absolute ruler at initialization, and forward-facing driving rarely revisits locations closely enough to trigger loops. Learning-based monocular geometry and metric-depth priors—including CNN-SLAM [10], MiDaS-style relative depth [53], ZoeDepth [54] (metric depth via relative + metric heads), DUSt3R [11], VGGT [12], MASt3R [44], MoGe/MoGe-2 [13], DINOv2-backed geometry [55], and long-sequence variants such as VGGT-Long [56] and MASt3R-SLAM [57]—provide dense geometry from RGB and are increasingly used inside monocular 3DGS-SLAM [36,37,39,40,42,43]. Even networks described as “metric” (ZoeDepth, MoGe-2, and MASt3R) retain residual global-scale bias outdoors and are not, by themselves, drop-in absolute-scale VO systems for SE(3); when fused into 3DGS pipelines, they often act as passive regularizers without an independently verifiable physical anchor, and published evaluations frequently lean on post hoc Sim(3) alignment [36,43], which is unavailable at deployment. Outdoor monocular 3DGS systems such as OpenGS-SLAM [42] and S3PO-GS [43] therefore improve appearance and coarse geometry yet leave absolute online metric consistency only partly solved under single-camera sensing. This gap sits within a wider autonomous-driving research landscape: the camera intrinsics assumed throughout our pipeline are themselves an active estimation target in recent learning-based calibration [58], while the metric maps we target are ultimately consumed by downstream perception and planning stacks, an area represented by multi-agent decision-making studies such as RSMIX [59].

Taken together, existing systems either recover the metric scale on a sparse back end, depend on an IMU or measured depth, or produce dense maps whose scale remains Sim(3)-ambiguous. MoGe2-SLAM is built for the remaining case: outdoor monocular 3DGS with an explicit camera-height and ground-plane prior. Absolute-Scale Initialization rescales the frozen MoGe-2 depth map on the first keyframe; Grid-Balanced Tracking and ORB-Anchored Mapping then carry that metric origin forward under weak outdoor texture, yielding online outdoor monocular 3DGS-SLAM in approximate physical units.

## 3. Materials and Methods

### 3.1. Problem Formulation and System Overview

Given a monocular RGB sequence {I_t_} with known intrinsics K, our goal is to estimate camera poses {T_t_} ⊂ SE(3) and a dense 3D Gaussian map 𝒢 = {(μ_i_, Σ_i_, α_i_, c_i_)} such that both the trajectory and the map are expressed in physical units (meters). Unlike indoor Gaussian SLAM, where revisits and structural regularity provide implicit scale cues, outdoor forward-facing driving offers long, largely non-looping trajectories dominated by weak texture and scale drift; MoGe2-SLAM therefore treats the metric scale as a quantity that must be explicitly initialized and continuously propagated, not recovered post hoc.

**Figure 2:**
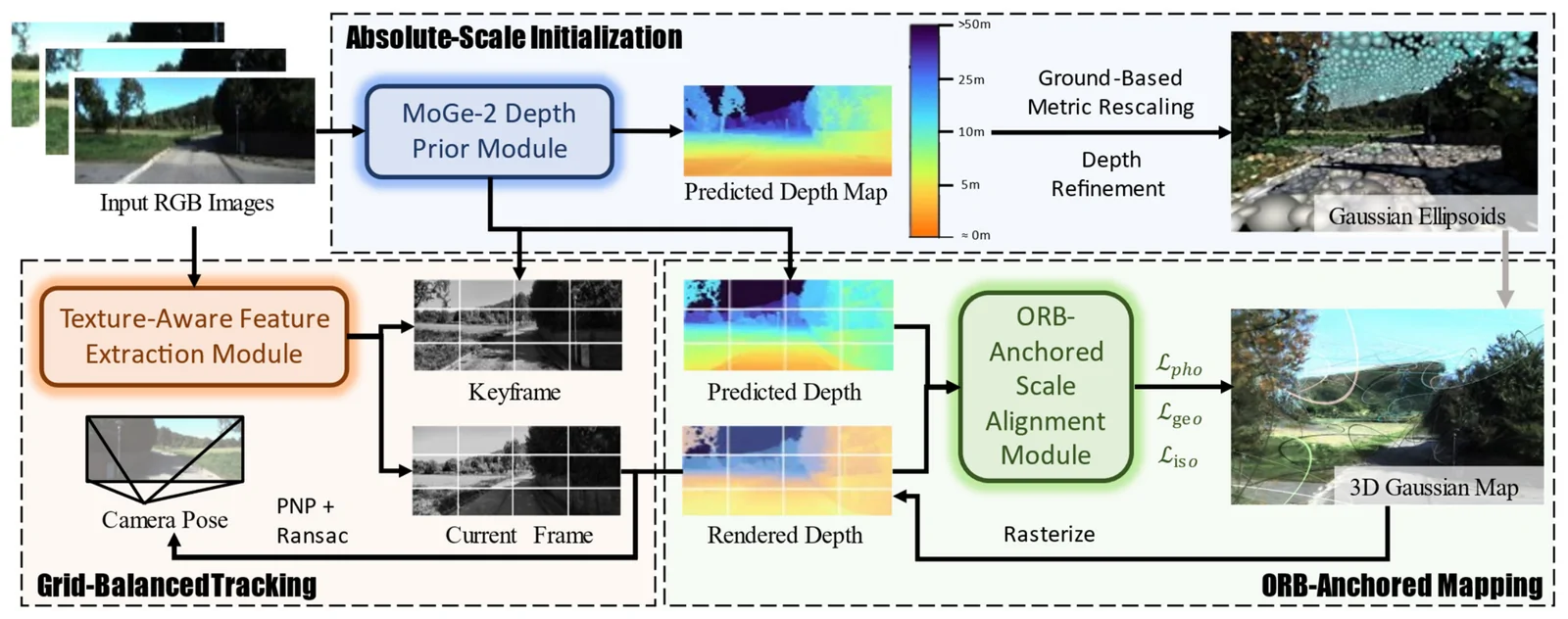
Overview of MoGe2-SLAM. Absolute-Scale Initialization establishes a metric depth origin; Grid-Balanced Tracking propagates stable poses under a weak outdoor texture; ORB-Anchored Mapping couples the evolving Gaussian map with the MoGe-2 prior to suppress long-horizon scale drift.

The proposed pipeline couples Absolute-Scale Initialization, Grid-Balanced Tracking, and ORB-Anchored Mapping into a single scale-consistency chain, as shown in Figure 2.

1. **Absolute-Scale Initialization** fixes a global physical ruler on the first keyframe by anchoring a frozen MoGe-2 depth prior to ground-plane geometry and a known camera height and then spawns the initial Gaussians from the resulting metric depth;
2. **Grid-Balanced Tracking** estimates a pose for every incoming frame from spatially balanced 2D–3D correspondences and a short photometric refinement against the current map;
3. **ORB-Anchored Mapping** operates on keyframes only, aligning rendered depth with the MoGe-2 prior through sparse ORB geometry and jointly optimizing poses and Gaussians in a sliding window.

The front end estimates a pose at sensor rate while holding the map fixed between mapper updates; the back end uses only keyframes to refine geometry, appearance, and per-keyframe depth-scale factors. After the native MoGe-2 global scale is replaced, these stages form an online metric SLAM loop without auxiliary sensors. Relative to prior monocular 3DGS-SLAM [36,42,43], the difference is how the scale is handled: it is (i) fixed by a physical camera–ground ruler before map birth, (ii) protected by spatially conditioned tracking so that poor 2D–3D geometry cannot quietly corrupt that ruler, and (iii) re-estimated as an online variable αk at every keyframe, rather than left to network self-consistency or post hoc Sim(3) alignment. Section 3.2, Section 3.3 and Section 3.4 detail each stage. Experiments in Section 4 show that MoGe2-SLAM achieves competitive tracking at an approximate absolute metric scale together with high-fidelity rendering, as shown in Figure 1.

### 3.2. Absolute-Scale Initialization

**Figure 3:**
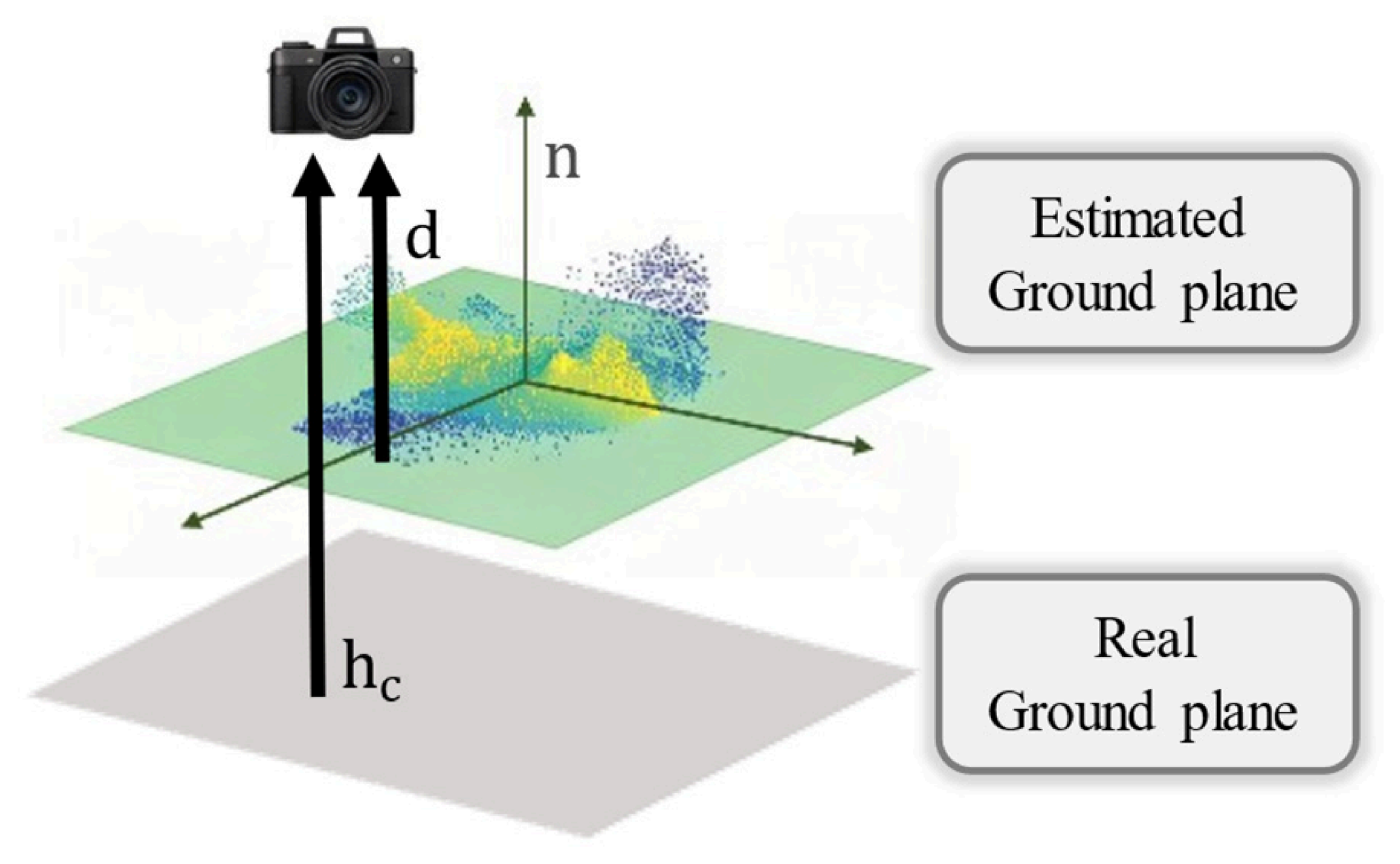
Absolute-Scale Initialization. MoGe-2 depth is rescaled by ground-plane geometry and camera height, refined, and back-projected to spawn metric Gaussians. The colored dots in the picture are schematic representations of abstract urban scene point clouds and do not represent real-world scenes.

Monocular geometry networks supply a dense structure, but a per-image metric factor is not automatically a SLAM-consistent absolute scale. Even though MoGe-2 [13] can predict metric-scale geometry, MoGe2-SLAM treats its frozen output as a dense geometric prior and makes the scale identifiable for outdoor SLAM on the first keyframe—before any Gaussian is created—by coupling that prior to a physical camera–road length under a local plane model that holds for typical outdoor driving. This design differs in kind from three common alternatives. Classical ground-plane visual odometry [46] recovers a trajectory scale after sparse triangulation but does not spawn a dense metric map that subsequent rendering can inherit. MonoGS [36] and related monocular 3DGS systems initialize Gaussians from relative triangulation or photometric bootstrapping, so the map is born under an unknown Sim(3). Prior-driven outdoor systems such as S3PO-GS [43] and OpenGS-SLAM [42] inject learned point maps or depths as dense supervision, yet without an explicit camera–ground ruler, their absolute scale remains weakly constrained for long non-looping drives. In contrast, Absolute-Scale Initialization overwrites MoGe-2’s global scale after the forward pass with dataset-wise camera-height calibration. MoGe-2 has no inference mode without its global scale head, so that head always runs. In our outdoor SLAM, however, the advertised global scale does not recover well; we therefore overwrite it with the camera-height calibration while retaining MoGe-2’s local geometry for map birth. MoGe-2 depth is rescaled by ground-plane geometry and camera height, refined, and back-projected to spawn metric Gaussians, as illustrated in Figure 3.

#### 3.2.1. Dense Depth Prior Interface

For the first keyframe I_0_, a frozen MoGe-2 network [13] predicts a dense depth map

D_0_ = F_MoGe-2_(I_0_), D_0_ ∈ ℝ^H×W^, (1)

which is back-projected to 3D samples X(u) = D_0_(u) K^−1^ũ for ũ = [u, v, 1]^T^. MoGe-2 [13] always runs its global scale head; there is no scale-head-off inference. We load the frozen checkpoint moge-2-vitl and consume only the dense depth/point-map pathway; the network is neither fine-tuned nor adapted online. MoGe-2 is a metric-scale method and always runs its global scale head (there is no scale-head-off inference). Since that advertised scale does not recover well on our outdoor protocol, the full system overwrites it with the camera-height calibration below.

#### 3.2.2. Ground-Plane Calibration

Absolute scale is identifiable once a single physical length is tied to the monocular reconstruction. Under the local road-plane model Π: n^T^X + d = 0 with ‖n‖_2_ = 1, the signed plane offset |d| is the camera-to-plane distance in the same units as the MoGe-2 depth; a calibrated mounting height h_c_ > 0 then uniquely determines a global similarity factor. Let 𝒳 be near-field ground candidates: points are drawn from a lower-image ROI (default: the lower 40% of the image height), not as an ad hoc preference but because rays that meet the road at a short range yield a better-conditioned estimate of d (larger angular support and higher depth SNR) than far-horizon samples. We estimate (n, d) by robust M-estimation after RANSAC (Random Sample Consensus),

min_(n,d)_ Σ_i∈𝒳_ ρ(n^T^ X_i_ + d) + λ_n_ (1 − n^T^ n_prior_), (2)

where ρ(·) is a robust penalty. The weight λ_n_ = 0.05 multiplies an optional Tikhonov term toward a gravity-aligned unit direction n_prior_. This n_prior_ is not a second required metric prior: the only external metric quantity required for Absolute-Scale Initialization is the known mounting height h_c_; the plane parameters (n, d) are estimated from near-field road points. The Tikhonov term, when enabled, merely discourages degenerate normals under sparse support. When no such soft cue is used, we set λ_n_ = 0. In all reported experiments we do not require IMU or gravity sensing. RANSAC removes dynamic objects and non-planar clutter before the M-step. The identified scale and calibrated depth are

s_0_ = h_c_^⊥^/|d|, D_0_^c^(u) = s_0_ D_0_(u), (3)

where h_c_^⊥^ is the plane-orthogonal camera–road distance implied by the vertical mounting height (see Section 3.2.3). The multiplicative map preserves MoGe-2’s local depth ordering while enforcing the physical camera–ground constraint; every subsequent Gaussian inherits this metric origin.

#### 3.2.3. Perturbation Analysis and Non-Ideal Ground

Because s_0_ is a ratio of two lengths, first-order relative errors propagate as

δs_0_/s_0_ ≈ δh_c_^⊥^/h_c_^⊥^ − δ|d|/|d|. (4)

A mounting-height tolerance of a few centimeters and a plane-offset residual of similar magnitude therefore contribute additively to relative scale error; neither term is amplified by scene depth. In practice, we quantify this uncertainty by bootstrap perturbation: M = 32 short RANSAC hypotheses are drawn by resampling 𝒳 with replacement and, optionally, by jittering h_c_ within ±0.02 m; the accepted scale is the median s_0_ = med_m_ {h_c_^⊥^/|d_(m)_|}, and the fit is rejected (triggering a wider ROI or a short wait for a clearer ground view) if the median absolute deviation satisfies MAD({s_0(m)_}) > 0.05 |s_0_|. This turns the initializer from a single-shot fit into a scale estimate with an explicit stability check.

Sloped but locally planar roads are already covered by the free normal n. Here, h_c_ is the vertical mounting height above the contact patch. The unit vector e_g_ is not an IMU reading; it is the nominal downward axis of a level camera mount, fixed in the camera frame. The plane-orthogonal distance used in (3) is h_c_^⊥^ = h_c_ |n^T^e_g_|; constant longitudinal or lateral grades therefore change n rather than invalidate the model. Mildly uneven or bumpy pavement appears as high-frequency residuals around a dominant support plane: RANSAC retains the largest consensus set, the M-estimator down-weights local undulations, and acceptance still hinges on the bootstrap dispersion above. Strongly non-planar patches (deep gutters and speed bumps filling the near field) reduce inlier support; when the residual RMS relative to |d| exceeds 0.10 (RMS/|d| > 0.10), we fall back to multi-patch planes in adjacent near-field cells and take a robust median of their scales. Any residual bias that survives initialization is not frozen forever: it is corrected online by the per-keyframe factors α_k_ in Section 3.4, which continuously re-align rendered depth with MoGe-2 under sparse ORB anchors.

#### 3.2.4. Depth Refinement and Gaussian Spawning

The calibrated depth D_0_^c^ is further refined to D_0_^r^ with an edge-preserving filter [60] that preserves object boundaries while suppressing high-frequency noise from the prior. Each valid pixel is converted to an anisotropic 3D Gaussian

G_i_(x) = exp(−½ (x − μ_i_)^T^ Σ_i_^−1^ (x − μ_i_)), (5)

with mean μ_i_ from back-projection, covariance Σ_i_ from local depth variation, and appearance parameters c_i_. Because D_0_^r^ is already metric, the initial map is born at approximate physical scale rather than at an arbitrary similarity transform.

### 3.3. Grid-Balanced Tracking

Open roads and the sky dominate many driving frames, causing standard feature detectors to cluster correspondences in textured facades while leaving large image regions unsupported. Degenerate 2D–3D configurations then destabilize PnP and amplify scale-sensitive drift. Grid-Balanced Tracking addresses this by enforcing a spatial budget of correspondences before pose estimation. The module is not a generic feature-balancing heuristic transplanted from classical SLAM. ORB-SLAM3 [19] redistributes keypoints primarily by detector response and local texture, which still starves homogeneous road cells that would otherwise contribute few pose constraints for forward driving. Purely photometric trackers such as MonoGS [36] refine the pose against a rendered map without an explicit spatial quota on 2D–3D matches, so early outdoor frames with biased correspondences can pull the metric map into locally consistent but globally skewed poses. Our occupancy-aware grid instead forces coverage before RANSAC-PnP and then uses a short photometric polish only as local correction, preserving the absolute-scale map inherited from Section 3.2 rather than re-learning the scale through appearance alone.

**Figure 4:**
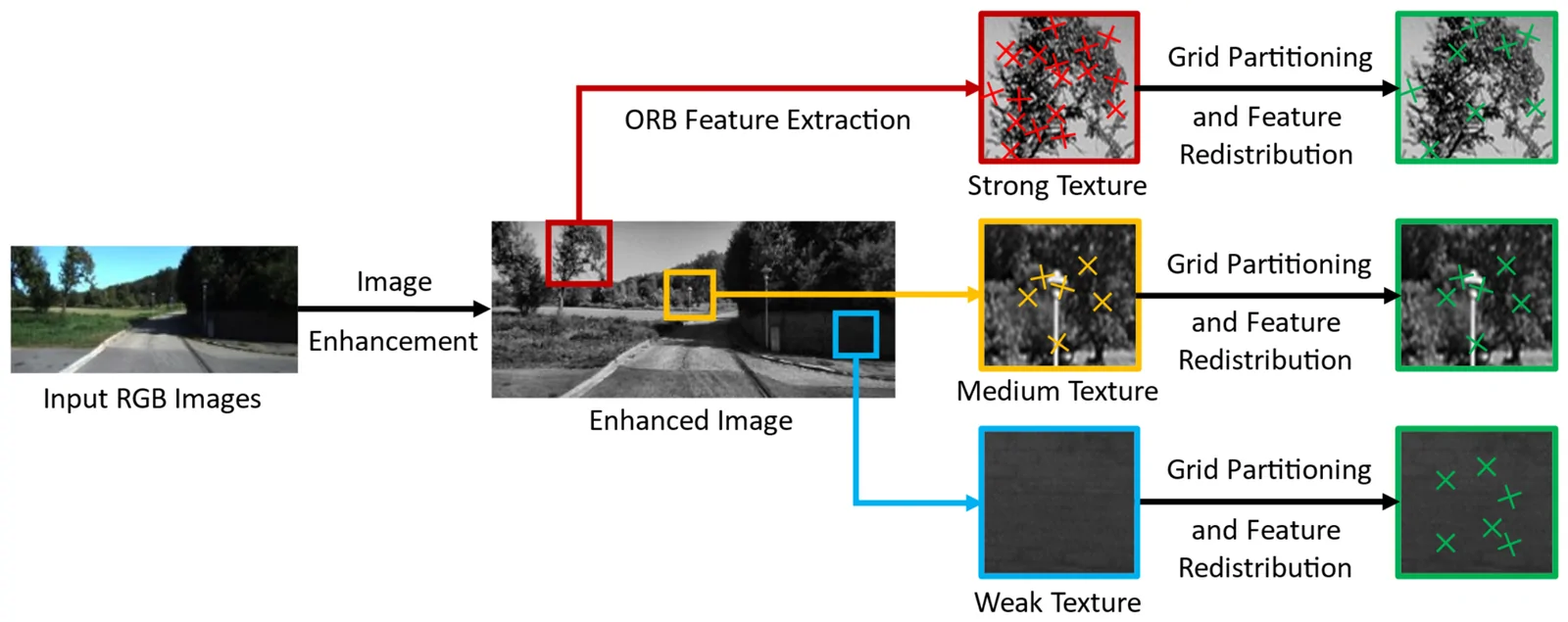
Grid-Balanced Tracking. Input RGB frames are enhanced, after which ORB features are extracted and redistributed on an occupancy grid so that high-texture facades cannot monopolize matches. Candidate balancing does not treat a homogeneous sky as a trusted geometric source: weighted RANSAC-PnP with geometric consistency retains only inliers for the pose. Colored boxes in the figure highlight regions of interest, while colored “×” marks denote key points.

Grid-Balanced Tracking redistributes correspondences on an occupancy grid before pose estimation, as illustrated in Figure 4. The image is partitioned into a G × G occupancy grid with cell size c_w_ = W/G and c_h_ = H/G. G is a relative split, not a fixed pixel window. All main experiments use G = 8 (8 × 8), chosen on KITTI-resolution clips by a short sweep over {6, 8, 10, 12, 16} and η ∈ {0.25, 0.5, 1.0} and then shared across KITTI/Waymo/Argoverse 2; G is retuned if the resolution changes substantially (Section 4.4.2). For a candidate i in cell g(i), a response-aware score with η = 0.5 (unless noted) penalizes overcrowded cells. Light Contrast-Limited Adaptive Histogram Equalization (CLAHE) and weak edge enhancement are applied prior to ORB detection so that road cells retain usable responses; sky detections are not used as trusted PnP constraints.

We do not fix a single FAST (Features from Accelerated Segment Test) threshold for all scenes, nor do we map a semantic texture class to τ_FAST_ by hand. Adaptation follows a texture proxy and the detection count. (i) On grayscale frame I, σ^2^ = Var(I). If σ^2^ < 800, an aggressive CLAHE/multi-scale edge path is used; otherwise, a milder path is used. Denoising is further reduced if the enhanced variance remains < 500. (ii) ORB is run with OpenCV fastThreshold τ_FAST_ = 5 and budget nfeatures = 8000. If fewer than 0.3 nfeature keypoints appear, we re-detect at τ_FAST_ = 3, and if fewer than 50 remain, at τ_FAST_ = 1; if the count is still below 200, a multi-scale bank with τ_FAST_ ∈ {8, 10, 12} is merged by response. (iii) Grid balancing then redistributes keypoints so high-texture regions cannot monopolize matches. Weighted RANSAC-PnP, not detection, decides the inlier set.

q_i_ = φ_i_/(1 + η N_g(i)_), η = 0.5, (6)

Here, φ_i_ is the detector response, and N_g(i)_ counts features already selected in cell g(i). High-response features in overcrowded cells are down-weighted, while under-populated cells are filled by borrowing from neighbors, yielding a predictable spatial footprint of matches before PnP. Compared with texture-driven redistribution as in ORB-SLAM3 [19], this fixed grid enforces coverage on homogeneous road regions where pose constraints would otherwise vanish. For match m, we assign weight w_m_ = c_m_ ξ_m_, combining descriptor confidence with geometric consistency. The pose is estimated by weighted RANSAC-PnP,

T_t_ = arg min_T∈SE(3)_ Σ_m∈ℐt_ w_m_ ρ(‖π(T P_m_) − u_m_‖_2_^2^), (7)

where P_m_ are 3D points lifted from the rendered depth of the last keyframe, u_m_ are observed features, and ℐ_t_ is the inlier set.

PnP yields a robust SE(3) pose from sparse 2D–3D matches but ignores dense appearance. Each frame is allocated up to 100 tracking iterations. We therefore refine only the pose T_t_ while keeping the Gaussian map 𝒢 fixed. Let I_render_ = I(𝒢, T_t_) denote the RGB image rendered from the current frozen map. Minimizing the photometric loss ℒ_track_, given by ℒ_track_ = 0.8 ‖I_render_ − I_t_‖_1_ + 0.2 (1 − SSIM(I_render_, I_t_)) in Equation (8), is the photometric polish: gradients flow through the rasterizer to the pose, while 𝒢 remains constant (Adam, five steps by default, learning rate 5 × 10^−3^).

ℒ_track_ = 0.8 ‖I_render_ − I_t_‖_1_ + 0.2 (1 − SSIM(I_render_, I_t_)). (8)

The iteration budget is adapted to tracking confidence: frames with fewer inliers receive more refinement steps, concentrating computation where geometric association is weakest. This hybrid design—sparse geometry for global alignment and dense rendering for local correction—is particularly important on long, straight segments where parallax alone is weak.

### 3.4. ORB-Anchored Mapping

The absolute physical scale is established only in Section 3.2; ORB-Anchored Mapping does not introduce a new camera–ground length. It propagates that initialized metric identity and corrects residual drift between the Gaussian map and the frozen MoGe-2 prior. The mapper refines 𝒢 and keyframe poses on a sliding window |𝒲| = 8, with up to 150 mapping iterations per keyframe. A frame becomes a keyframe when translation exceeds 0.08 $\hat{D}$/0.05 $\hat{D}$ ($\hat{D}$ = median rendered depth), overlap falls below 0.9, tracking quality degrades, or five frames have elapsed, following MonoGS [36] and S3PO-GS [43]. Unlike photometric-only MonoGS or MASt3R-supervised S3PO-GS, we treat αk as an online drift-correction state tied to sparse ORB landmarks, injecting scaled prior detail only where the map disagrees.

#### 3.4.1. Rendering Model

For each pixel ray u, color and depth are obtained by alpha compositing over sorted Gaussians:

(9) $$\hat{I}(u)=\Sigma_{i}T_{i}\alpha_{i}c_{i},\hat{D}(u)=\Sigma_{i}T_{i}\alpha_{i}d_{i},T_{i}=\Pi_{j<i}(1-\alpha_{j}).$$

Because 𝒢 is initialized in metric units, rendered depth $\hat{D}$ lives in the same physical frame as the MoGe-2 prior.

#### 3.4.2. ORB-Anchored Depth-Scale Alignment

**Figure 5:**
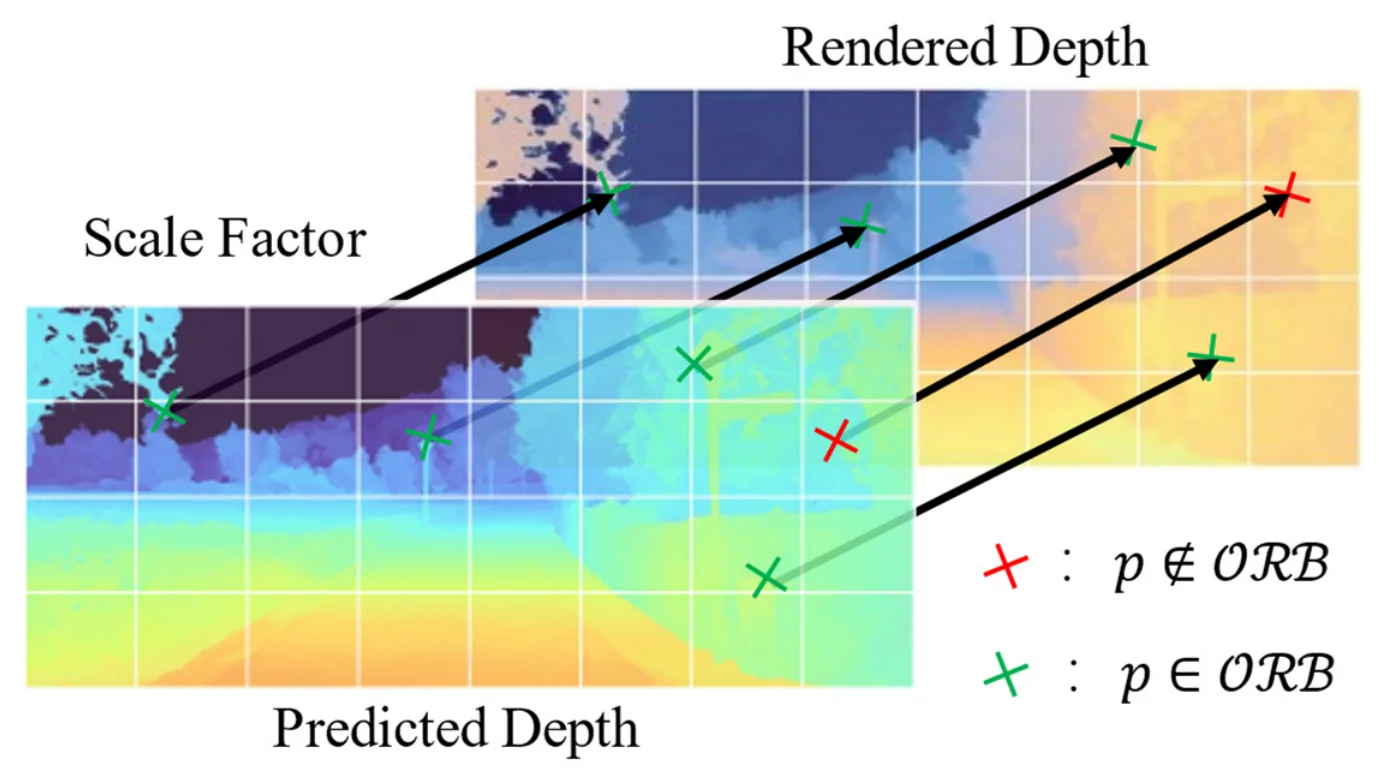
ORB-Anchored Mapping. Sparse ORB anchors couple rendered depth with the MoGe-2 prior; per-keyframe scale factors are optimized jointly with photometric and geometric losses in a sliding window.

MoGe-2 provides sharp local structure but can drift in global scale across keyframes; rendered depth carries the current map scale but may blur fine detail. We therefore align the two sources at each keyframe k through sparse ORB anchors {X_j_} with observations u_k,j_, as illustrated in Figure 5. For each keyframe, we estimate a positive scale α_k_ relating prior depth D_k_^p^ to rendered depth $\hat{D}$_k_:

(10) $$r_{k}(u)={\hat{D}}_{k}(u)-\alpha_{k}{D_{k}}^{p}(u),\;{ẽ}_{k}(u)=|r_{k}(u)|/(\alpha_{k}{D_{k}}^{p}(u)+\varepsilon ).$$

The scale α_k_ is updated iteratively by retaining pixels with small relative error ẽ_k_ and recomputing a robust ratio of means between $\hat{D}$_k_ and D_k_^p^; when dense agreement is insufficient, α_k_ is re-seeded from ORB correspondences between consecutive keyframes (still a relative alignment step, not a new physical calibration). Pixels where rendered and scaled prior depth disagree beyond a tolerance are replaced by α_k_ D_k_^p^, injecting prior detail into regions where the map is still immature. Sparse ORB anchors are essential to this step: unlike dense prior–render residuals alone, they provide viewpoint-stable geometric ties that keep α_k_ observable when photometric agreement is ambiguous. The resulting alignment is therefore best read as preserving and correcting scale drift relative to the initialized metric map: ORB anchors make α_k_ observable, but they do not themselves supply a new absolute length.

#### 3.4.3. Sliding-Window Optimization

The mapper minimizes

ℒ_map_ = Σ_k∈𝒲_ (λ ℒ_k_^pho^ + (1 − λ) ℒ_k_^geo^) + λ_iso_ ℒ_iso_, (11)

with photometric loss ℒ_k_^pho^ = ‖I(𝒢, T_k_) − I_k_‖_1_, geometry loss ℒ_k_^geo^ = ‖X_k_^r^ − $\hat{X}$_k_^p^‖_1_ between rendered and scale-aligned prior point maps [46], and isotropic regularization ℒ_iso_ = Σ_i_ ‖s_i_ − $\overset{-}{s}$_i_ 1‖_1_ that discourages needle-like Gaussians under sparse views. In all experiments, we set λ = 0.98 and λ_iso_ = 10 in Equation (11) so that photometric fidelity dominates while a weak geometry term and a moderate isotropic penalty stabilize the map. Unlike methods that report trajectory shape under Sim(3) alignment, MoGe2-SLAM estimates {α_k_} during online mapping. The initialization ruler (Section 3.2) fixes the origin of scale; Grid-Balanced Tracking (Section 3.3) keeps pose estimation well conditioned; ORB-anchored alignment propagates and corrects the scale at every keyframe. This closed loop is the central design principle of the method.

## 4. Results

### 4.1. Experimental Setup

**Datasets.** We evaluate on 27 outdoor driving clips, 9 from each of KITTI Odometry [61], Waymo Open Dataset [62], and Argoverse 2 [63]. Unless a subsection studies a smaller controlled subset, every main ATE/novel-view/path-length mean uses these same 27 clips. Exact sequence IDs, inclusive frame ranges, and clip lengths are listed in Table S1 of the Supplementary Materials.

**Metrics and reporting.** We report tracking accuracy using the root-mean-square Absolute Trajectory Error (ATE) on keyframes [64], evaluated under two alignment protocols. Under Sim(3) alignment, we solve for the optimal similarity transform [65] that best aligns the estimated trajectory to ground truth:

ATE-Sim(3) = √(1/N Σ_i_ ‖t_i_^gt^ − (s R t_i_^est^ + t)‖_2_^2^), (12)

with scale s left free; ATE-Sim(3) mainly reflects trajectory shape fidelity. Under SE(3) alignment, we fix the scale to 1 and solve only for rigid rotation and translation:

ATE-SE(3) = √(1/N Σ_i_ ‖t_i_^gt^ − (R t_i_^est^ + t)‖_2_^2^), (13)

so that any systematic or drifting metric bias manifests directly in the reported error. ATE-SE(3) is therefore the metric that matches our absolute-scale design goal most closely. We additionally report (i) per-sequence relative scale error εs = |ρ − 1|; (ii) segment-wise drift; (iii) translational/rotational Relative Pose Error (RPE); and (iv) scale error versus traveled distance. Novel-view Peak Signal-to-Noise Ratio (PSNR), SSIM [66], and Learned Perceptual Image Patch Similarity (LPIPS) [67] follow the held-out non-keyframe protocol of Section 4.3. (keyframes used for mapping are excluded), with identical evaluation views for all NeRF/3DGS methods.

**Baseline methods and selection rationale.** We compare against the main outdoor monocular mapping approaches. (i) Outdoor RGB-only monocular 3DGS-SLAM: MonoGS [36], OpenGS-SLAM [42], S3PO-GS [43], and VINGS-Mono [41] in monocular mode with the IMU disabled. (ii) Dense neural mapping: NeRF-SLAM [33] and NICER-SLAM [34]. (iii) Sparse/feed-forward geometry: ORB-SLAM3 [19] and VGGT-Long [56]. (iv) Absolute-scale monocular VO controls (no IMU): ORB-SLAM2-HScale [46] and LIBVISO2 Mono [45]. These VO rows are the fair SE(3) references for the camera-height prior. Novel-view metrics are reported only for NeRF/3DGS methods. Large SE(3) gaps versus ruler-free 3DGS/NeRF entries are partly expected; Sim(3) remains the fairer shape ranking across unequally informed systems. All experiments run on a single NVIDIA RTX A6000 GPU.

**Camera-height prior.** Absolute-Scale Initialization uses a fixed vertical camera mounting height h_c_ as the external metric prior. Unless otherwise stated, we set h_c_ = 1.65 m on KITTI [61], h_c_ = 2.12 m on Waymo Open Dataset [62] FRONT, and h_c_ = 1.38 m on Argoverse 2 [63] ring_front_center. These values are held constant within each dataset; we do not claim operation without this metric prior.

Monocular 3DGS-SLAM shows modest run-to-run variation due to RANSAC sampling, keyframe scheduling, and optimizer initialization. Unless noted otherwise, MoGe2-SLAM entries in the table below report per-clip means ± sample standard deviations (SDs) over ten independent trials with identical hyperparameters and hardware. Dataset-level means average the per-clip means, and the reported ± values average the per-clip sample SDs. Published KITTI/Waymo 3DGS numbers and the camera-height VO controls remain single-number rows.

### 4.2. Scale Recovery and Camera Tracking

#### 4.2.1. Tracking ATE Under Sim(3) and SE(3)

We report keyframe ATE under Sim(3) and SE(3) (Table 1, Table 2 and Table 3; MoGe2-SLAM as ten-run mean ± SD). Sim(3) evaluates trajectory shape with a free global scale and is the fairer ranking when a baseline has no external ruler. SE(3) keeps the scale fixed, so the score mixes tracking quality with whatever metric information the system actually uses. MoGe2-SLAM uses a known camera height; most listed monocular 3DGS methods do not. ORB-SLAM2-HScale [46] (HScale for short) and LIBVISO2 Mono [45] are the matched camera-height VO controls.

On KITTI (Table 1), ORB-SLAM3 is strong in shape (0.890 m Sim(3)) but weak in metric units (38.963 m SE(3)) when loops are scarce. Under the same RGB-only protocol, S3PO-GS (1.106 m Sim(3); 25.129 m SE(3)) and VINGS-Mono (IMU off) (1.024 m Sim(3); 37.242 m SE(3)) show the same pattern. MoGe2-SLAM reports 0.747 ± 0.356 m Sim(3) and 2.583 ± 0.631 m SE(3): Sim(3) stays with the stronger 3DGS entries, while SE(3) improves once the camera-height prior is used. Most monocular 3DGS baselines in Table 1, Table 2 and Table 3 do not receive that ruler, so a large SE(3) gap relative to those methods confounds tracking quality with unequal metric information. Matched-sensor SE(3) comparisons are therefore made against ORB-SLAM2-HScale [46] and LIBVISO2 Mono [45], which share the camera-height prior: SE(3) is lower (2.583 m versus 17.31 m/24.85 m), and the reconstructed map is dense rather than sparse.

Waymo and Argoverse 2 show the same split, with larger overall drift (Table 2 and Table 3). Sim(3) remains competitive: on Waymo, MoGe2-SLAM reaches 0.693 ± 0.135 m, third after ORB-SLAM3 (0.419 m) and S3PO-GS (0.622 m); on Argoverse 2, it reaches 0.510 ± 0.163 m. SE(3) is 5.463 ± 0.327 m on Waymo, comparable to ORB-SLAM2-HScale [46] (5.134 m); the margin over the matched-prior VO control seen on KITTI and Argoverse 2 disappears here, consistent with the larger, more distance-dependent scale shortfall on Waymo reported in Table 4 (ρ = 83.42%; ε_s_ = 0.166). On Argoverse 2, SE(3) is 3.920 ± 0.889 m, below ORB-SLAM2-HScale [46] (6.439 m).

**Figure 6:**
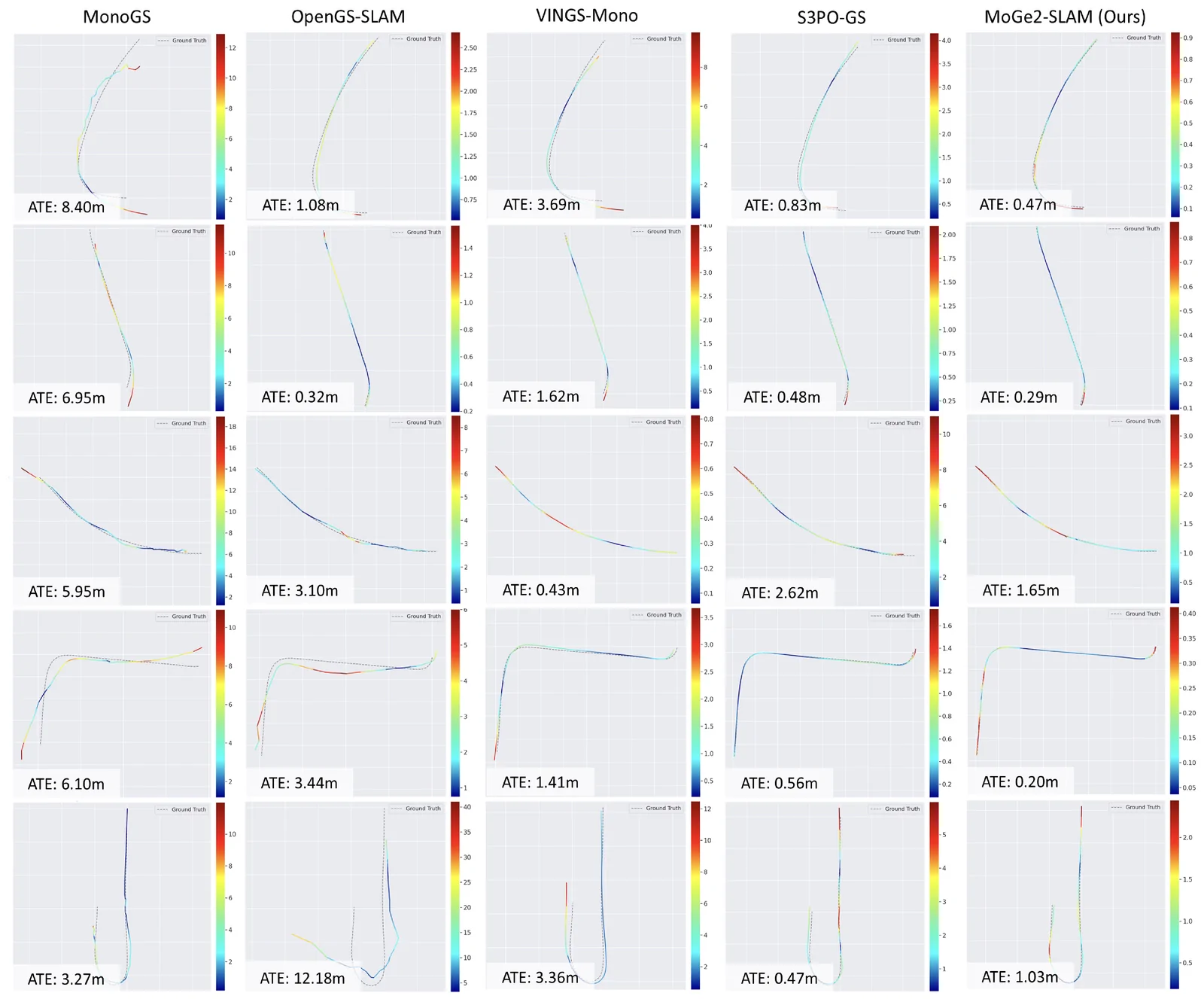
Estimated trajectories compared with ground truth after Sim(3) alignment for MoGe2-SLAM and representative 3DGS baselines on selected KITTI, Waymo, and Argoverse 2 sequences. Sim(3) absorbs a global similarity and therefore emphasizes trajectory-shape fidelity rather than the absolute metric scale.

The selected trajectories, after Sim(3) alignment, mainly reflect shape agreement with the ground truth, as shown in Figure 6. Because the global scale is free, methods without a metric scale may appear closer to the ground truth here than under SE(3).

**Figure 7:**
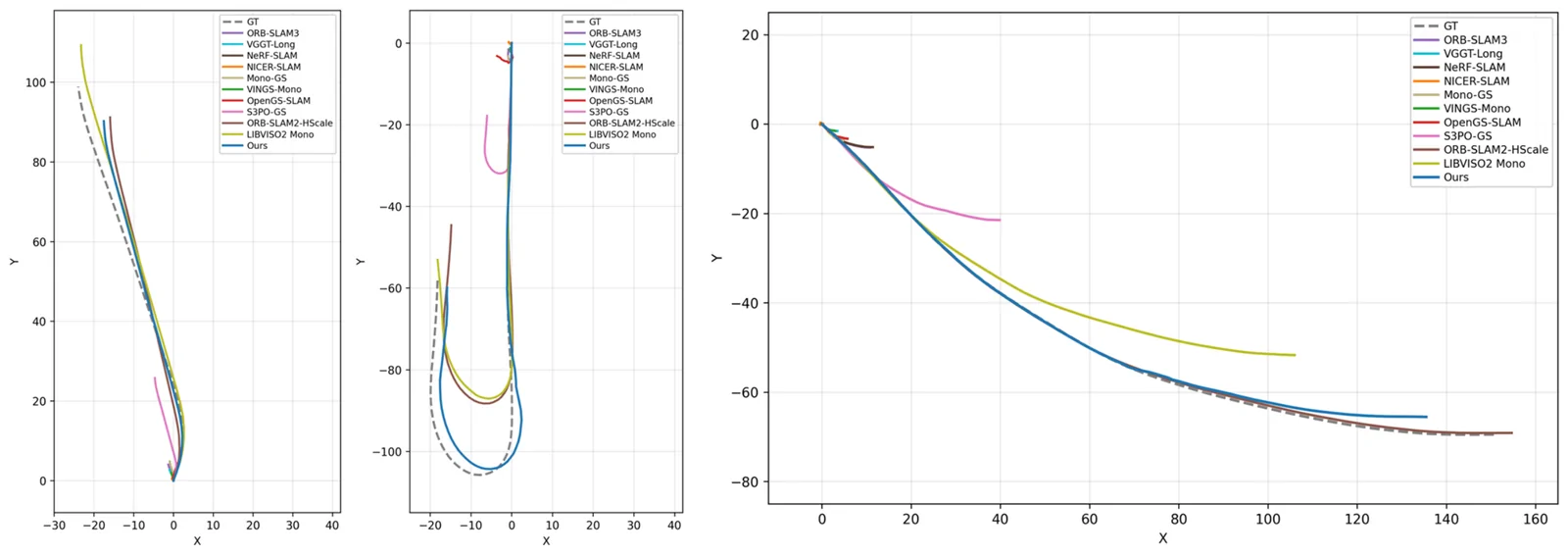
SE(3)-aligned trajectories of MoGe2-SLAM and competing methods against ground truth. MoGe2-SLAM (dark blue) tracks the reference path (gray dashed), while baseline estimates (other colors) deviate mainly in the overall metric scale.

Under strict SE(3) alignment, MoGe2-SLAM follows the ground-truth path more closely in metric units, whereas several baselines show clear scale discrepancies, as shown in Figure 7. The dark blue solid curve (MoGe2-SLAM) closely tracks the gray dashed ground truth, while the other colored curves (baselines) are visibly offset. This provides direct visual evidence that, among existing monocular SLAM methods, MoGe2-SLAM more faithfully preserves metric scale without additional sensors.

#### 4.2.2. Fine-Grained Metric-Scale Diagnostics

We summarize dataset-level mean ρ, relative scale error ε_s_ = |ρ − 1|, translational/rotational RPE, and the mean maximum segment-wise deviation |ρ(k) − 1| with ΔL = 50 m in Table 4, where ρ(k) is the path-length ratio on the k-th consecutive 50 m ground-truth segment. Over the 27-clip protocol, ρ = 90.17% (KITTI 93.37%, Waymo 83.42%, and Argoverse 2 93.72%), and ε_s_ = 0.103. Neighboring 50 m segments are consistent with one another (mean std. of ρ_(k)_ ≈ 0.014), yet the trajectory remains about 10% short (mean max |ρ_(k)_ − 1| ≈ 0.116). Mean RPE is 0.21 m/0.15° on KITTI, 0.45 m/0.17° on Waymo, and 2.67 m/2.06° on Argoverse 2. Scale-versus-distance curves stay below ρ = 1: KITTI remains near 0.92–0.94 out to 200–300 m, whereas Waymo falls from 0.84 (0–50 m) to 0.73 (200–300 m), and the pooled ratio from 0.91 to 0.86. The camera-height ruler therefore recovers absolute-scale trajectories under SE(3) without post hoc scaling, with a residual path-length shortfall that is larger and more distance-dependent on Waymo. Per-sequence scale diagnostics and prefix scale-error curves are reported in Table S8 and Figure S1 of the Supplementary Materials.

### 4.3. Novel-View Rendering

For each clip, we fix in advance a common set of non-keyframe evaluation frames by index; these frames are excluded from map optimization for every NeRF/3DGS method, regardless of that method’s own keyframe schedule. PSNR, SSIM [66], and LPIPS [67] are averaged only over the held-out frames, after the final map has been frozen, at the native calibration resolution. Dynamic masks, when used, affect training only. Fixing evaluation views by frame index keeps the comparison matched when keyframe policies differ. Point-map-only baselines are omitted because they do not produce a comparable dense render. Under the held-out novel-view protocol, mapping quality on KITTI, Waymo, and Argoverse 2 is compared against NeRF/3DGS baselines, as shown in Table 5, Table 6 and Table 7.

On KITTI, MoGe2-SLAM reports PSNR/SSIM/LPIPS of 21.29 dB/0.70/0.30, compared with 20.01 dB/0.64/0.41 for S3PO-GS. On Waymo, S3PO-GS attains a slightly higher PSNR (26.72 vs. 26.68 dB), while MoGe2-SLAM is better on SSIM (0.85 vs. 0.84) and LPIPS (0.31 vs. 0.36). On Argoverse 2, PSNR and SSIM are essentially tied with S3PO-GS (25.87 vs. 25.83 dB; SSIM 0.76/0.76), and MoGe2-SLAM is better on LPIPS (0.45 vs. 0.49). Overall, MoGe2-SLAM is best on all three metrics on KITTI; on Waymo and Argoverse 2, it ranks first or second on PSNR, first or tied on SSIM, and is best on LPIPS on both.

**Figure 8:**
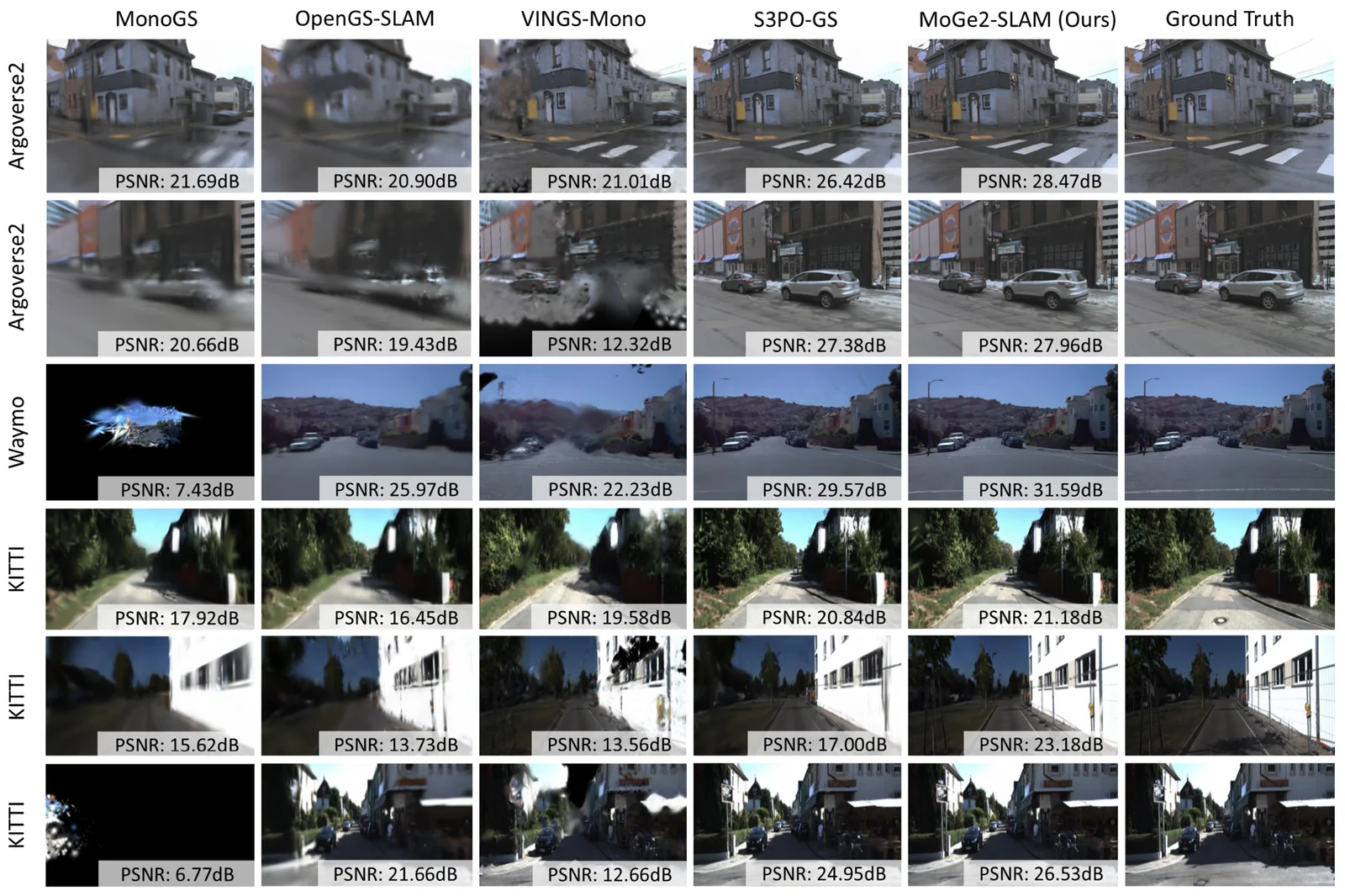
Qualitative comparison of novel-view synthesis on representative KITTI, Waymo, and Argoverse 2 scenes. MoGe2-SLAM produces photorealistic images with sharper geometric boundaries and fewer floaters than competing monocular 3DGS baselines.

Qualitative novel-view results across KITTI, Waymo, and Argoverse 2 are provided, as shown in Figure 8. In challenging outdoor sequences, pure monocular 3DGS baselines often exhibit severe blur, tracking-induced artifacts, and spurious floaters, whereas MoGe2-SLAM maintains stable novel views with sharper building silhouettes and fewer floaters, consistent with its leading or near-leading PSNR/SSIM/LPIPS.

**Figure 9:**
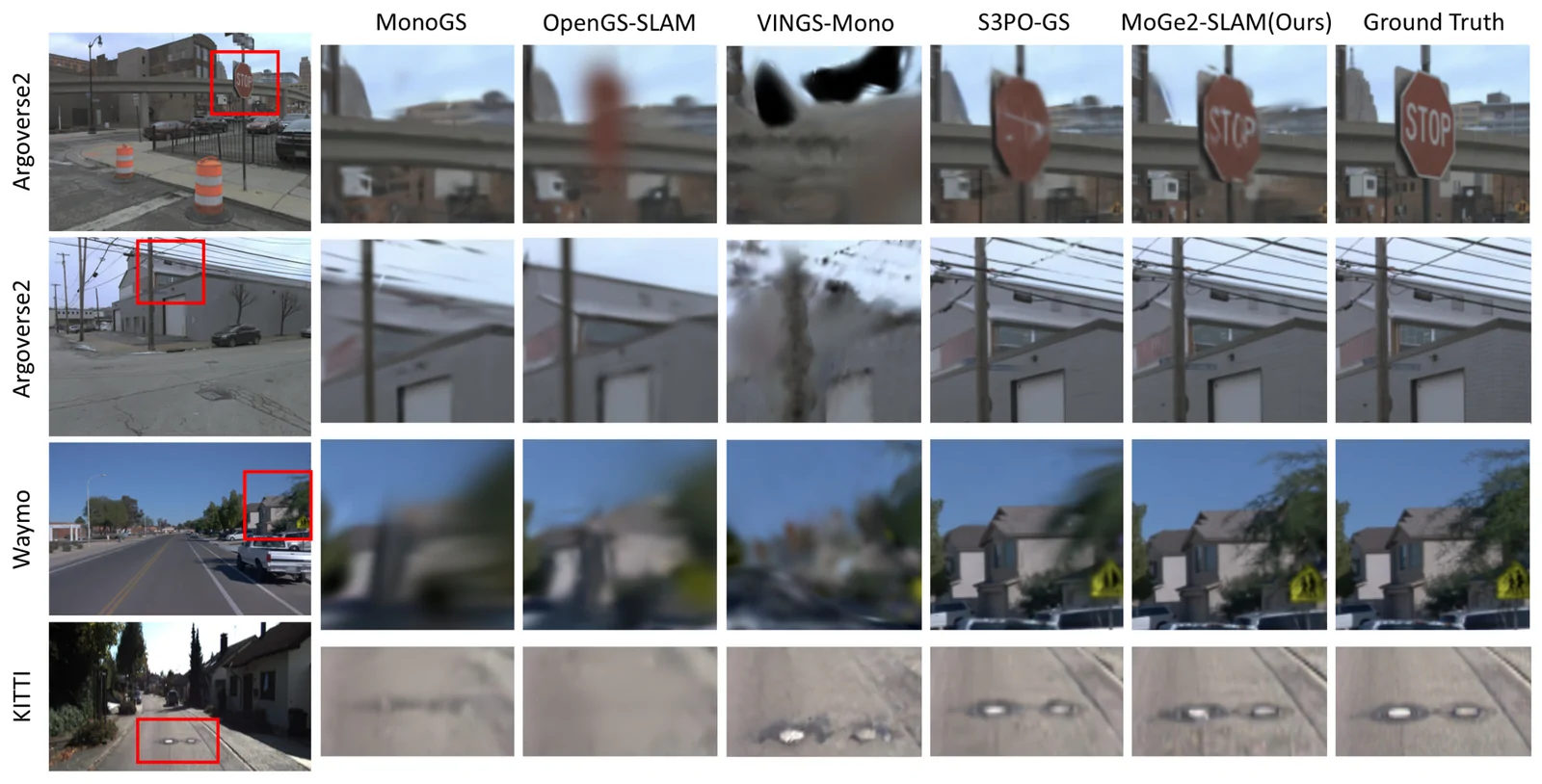
Visual comparison of local details. The leftmost column shows the reference view with the region of interest highlighted; subsequent columns show zoomed-in patches rendered by competing methods. MoGe2-SLAM recovers finer geometric structures and textures that more closely match the ground-truth appearance.

Local details are further examined in zoomed-in patches, as shown in Figure 9. MoGe2-SLAM better preserves high-frequency structure (sign lettering, thin poles, vehicle edges, and road texture) where competing methods over-smooth or stretch textures once scale and geometry become inconsistent. These qualitative observations align with the quantitative gains in Table 5, Table 6 and Table 7.

### 4.4. Ablation Study

#### 4.4.1. Module Ablation

**Figure 10:**
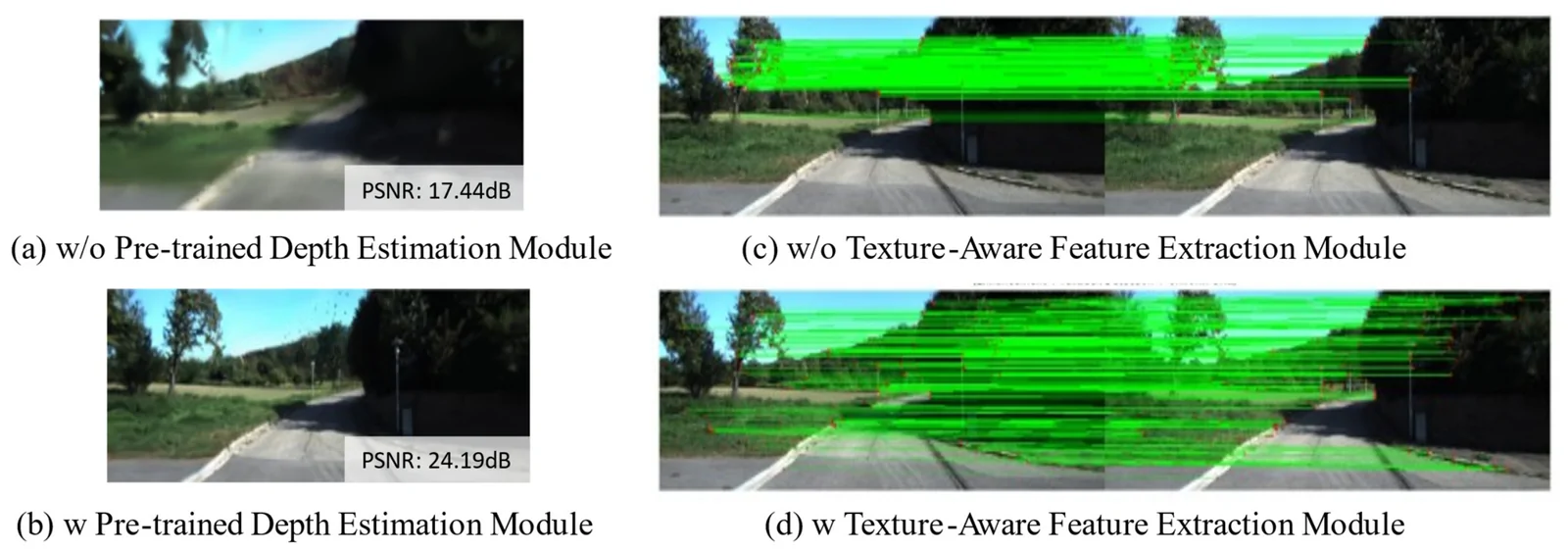
Ablation of the MoGe-2 depth prior and Grid-Balanced Tracking. (**a**) Without the prior, outdoor reconstructions become over-smoothed with severe geometric artifacts (example: PSNR 17.44 dB). (**b**) With the prior, Absolute-Scale Initialization and metric depth cues restore structural fidelity (example: PSNR 24.19 dB). (**c**) Without occupancy-aware grid balancing, correspondences collapse onto high-texture facades, while weak-textured roads remain unsupported. (**d**) With grid balancing, matches cover the image more uniformly, stabilizing PnP in outdoor driving scenes.

We compare the dense prior, the camera-height ruler, Grid-Balanced Tracking, and ORB-Anchored Mapping on the nine KITTI clips in Table 8. Every variant is worse than MoGe2-SLAM (Ours) on all five metrics. For clarity, we name the prior/ruler rows by what they run: MASt3R w/ camera height, MASt3R w/o camera height, and MoGe-2 w/o camera height. Under matched “no camera-height” conditions, replacing MASt3R by MoGe-2 yields the largest SE(3) reduction (MASt3R w/o camera height 15.104 m → MoGe-2 w/o camera height 5.944 m), indicating that the MoGe-2 geometric prior—not camera height alone—accounts for most of the gain. Enabling camera height on MoGe-2 then further lowers SE(3) from 5.944 m to 2.583 m; the MoGe-2 w/o camera height versus Ours comparison is the camera-height ablation on the intended prior. MASt3R w/ camera height is worse than MASt3R w/o camera height (16.952 m versus 15.104 m). Absolute-Scale Initialization presumes the prior depth differs from metric depth by a single global factor; MoGe-2 satisfies this, but MASt3R does not, so its fitted s_0_ is unreliable, and rescaling can even increase disagreement with the road plane. The wrong metric origin is then inherited by the first keyframe and preserved downstream. That row is therefore a prior-swap control rather than evidence against camera-height calibration. The same prior gap is visible qualitatively between runs without and with the MoGe-2 prior, as shown in Figure 10. Removing Grid-Balanced Tracking weakens pose estimates (1.392/3.864 m versus 0.747/2.583 m). Removing ORB-Anchored Mapping raises SE(3) to 4.706 m and gives the weakest novel-view scores (PSNR 20.41 dB; LPIPS 0.38). Both modules reuse the densification and photometric optimization skeleton shared with S3PO-GS [43]; we do not claim a new generic optimizer. Their role here is to protect and propagate the camera-height metric origin under outdoor weak texture, alongside the MoGe-2 prior. Additional prior-swap and cross-dataset ablation sheets are given in Tables S9–S11 of the Supplementary Materials.

#### 4.4.2. Controlled Ablation of Grid-Balanced Tracking

For the weak-texture 200-frame window in KITTI 03, we summarize how occupancy-grid coverage, correspondence count, and ATE vary with sampling and enhancement settings in Table 9. Conventional ORB has a high inlier ratio, but its matches cluster in a few textured patches, so occupancy-grid coverage is low; we therefore use an occupancy grid to spread matches. Coverage rises once sampling accounts for empty cells, with G trading uniformity against the count: G = 6 is the most uniform but the sparsest, G = 16 restores many matches but falls below conventional ORB in coverage, and the default G = 8 (Full Grid-Balanced Tracking) lies between the extremes in both coverage and count. Uniform-grid sampling without the response-aware score improves coverage but yields fewer matches. Full Grid-Balanced Tracking gives the lowest Sim(3) here, though SE(3) is slightly lower at G = 6, so G = 8 is an operating point rather than a winner in every column; the occupancy grid supplies the weak-texture sampling prior, and Table 8 remains the evidence that Grid-Balanced Tracking is needed in the full system.

### 4.5. Camera-Height Calibration Sensitivity

Equation (4) in Section 3.2.3 is a first-order relation for the relative scale at initialization. Table 10 is not a direct plot of that relation. It reports end-to-end ATE-SE(3) on short clips after an incorrect height or a geometric perturbation has passed through tracking and mapping, together with ρ and ε_s_ = |ρ − 1| from the same trajectories before SE(3) alignment. On KITTI 00, 05, and 07, we inject height errors δh_c_ ∈ {−15, −10, −5, 0, +5, +10, +15}%. On KITTI 07, we additionally test synthetic road tilt, uneven ground, camera pitch/roll, and partial occlusion of the near-field ground ROI (Table 10). All runs use the same short-clip windows as the main protocol for those sequences.

Initialization is never rejected in these tests. For incorrect h_c_, the mean ATE over 00/05/07 is 0.62 m at the nominal height and peaks near δh_c_ = −10% (0.81 m); the curve is not monotone (δh_c_ = −15% gives 0.55 m). The mean ρ stays between about 0.856 and 0.881 across the ±15% height errors (ε_s_ ≈ 0.12–0.14), so the path scale shifts only mildly once tracking and mapping have run. Geometric perturbations are reported on KITTI 07 only, where the unperturbed ATE is about 0.33–0.34 m and ρ stays near 0.91–0.92 (ε_s_ ≈ 0.08–0.09). The stronger ATE effects are +5° pitch (0.54 m) and 50% ground-ROI occlusion (0.51 m); small tilt (0.34 m) and uneven ground with σ = 0.02–0.10 m (0.36 m) remain mild, with ρ essentially unchanged relative to the nominal KITTI 07 clip. Table 10 should therefore be read as an operating envelope: the ATE stays in 0.53–0.81 m and is not monotone in |δh_c_|, while ρ is the more direct scale measure. Moderate ground-plane error has limited impact on both the ATE and ρ, but a badly wrong height is not harmless. We retain the known-h_c_ assumption and treat these numbers as its envelope on KITTI.

### 4.6. Robustness Analysis

Main outdoor benchmarks often emphasize well-conditioned clips. To check selection bias in the 27-clip protocol, we add an eight-clip hard-case suite that is not folded into Table 1, Table 2, Table 3, Table 4, Table 5, Table 6 and Table 7. It covers dynamic traffic, weak parallax, occlusion, and illumination change (Table 11). Six windows are new; Waymo 13476 and 153495 reuse FRONT logs from Table 2 and Table 5 because they concentrate traffic and lighting stress. A hard fail means tracking is lost and no trajectory is produced. Finishing with a large ATE is reported as accuracy degradation, not as a hard fail. A run whose estimated path is far too short for a driving window is marked as degenerate and is not counted as a successful metric result. Under the same clip list, we also evaluate S3PO-GS [43] as a strong outdoor monocular 3DGS baseline.

MoGe2-SLAM finishes seven out of the eight windows. The occlusion window Argoverse 2 30e94a6b is degenerate and is not treated as a success. Weak-parallax highway motion remains the accuracy failure: KITTI 01 reaches 8.42 m ATE-SE(3) (local error about 20 m), while the paired late window of KITTI 07 is milder (1.47 m). The usable occlusion clip 4612f4a4 reaches 0.46 m. The dynamic-traffic clips finish at 0.29 m (Argoverse 2 48c9cd36) and 0.34 m (Waymo 13476); the illumination clips at 1.65 m (Waymo 153495) and 1.35 m (KITTI 07, 200–399). Tracking is therefore usually retained. Across the eight stress windows, MoGe2-SLAM is better than S3PO-GS on both weak-parallax windows, on both illumination windows, and on the Waymo dynamic-traffic window, while S3PO-GS is slightly better on the Argoverse 2 dynamic-traffic window (0.21 m vs. 0.29 m) and on the usable occlusion window (0.17 m vs. 0.46 m). Both methods fail on the same degenerate window, indicating that the difficulty is intrinsic to that clip rather than method-specific.

### 4.7. Latency, Memory, Map Growth, and Extended-Sequence Stress Test

We profile the latency, peak memory, and active map size on KITTI 07 with the same protocol as MonoGS and S3PO-GS on one NVIDIA RTX A6000. We report the mean per-frame tracking time, mean keyframe mapping time, peak GPU memory, and number of active Gaussians |G| at a fixed late-frame checkpoint. MoGe-2 depth maps are precomputed and cached, so we measure only the online SLAM loop in Table 12. Separately, on the same RTX A6000, frozen MoGe-2 ViT-L inference takes about 55 ms per KITTI-resolution image under FP16 (mean 54.5 ms over 50 synchronized runs on a 376 × 1241 frame), consistent with the ≈50–60 ms latency reported by the model authors on A100/RTX 3090-class GPUs [13]; we treat this as offline prior preprocessing and do not include it in Trk or Map. Tracking throughput is summarized as 1/Trk (Hz); keyframe mapping remains asynchronous with respect to every camera frame.

A clear accuracy–cost trade-off is reported in Table 12. MoGe2-SLAM uses slightly more tracking and mapping time than MonoGS but records the lowest peak memory and the smallest active Gaussian count among the compared splatting systems. The mean tracking time is 0.961 s/frame (≈1.04 Hz) versus 0.643 s for MonoGS and 1.028 s for S3PO-GS; the mean mapping time is 2.030 s/keyframe versus 1.053 s/1.922 s; peak GPU memory is 8.2 GB versus 9.0 GB/9.5 GB; and active |G| is 1.768 × 105 versus 1.806 × 105/1.955 × 105. Relative to MonoGS, the extra cost comes mainly from denser geometric constraints (ORB-Anchored depth alignment and scale-aware densification) rather than from a larger primitive budget. Relative to S3PO-GS, MoGe2-SLAM is slightly faster in tracking, similar in mapping, and leaner in memory and |G|. The smaller |G| is consistent with geometry-constrained densification: ORB-anchored fusion favors depth-consistent regions and reduces compensatory clone/split and transient floaters that grow under appearance-only updates. On 10 Hz KITTI sensing, this profile is not real-time; it targets offline/near-online research evaluation with a lower-memory accuracy–cost trade-off.

**Figure 11:**
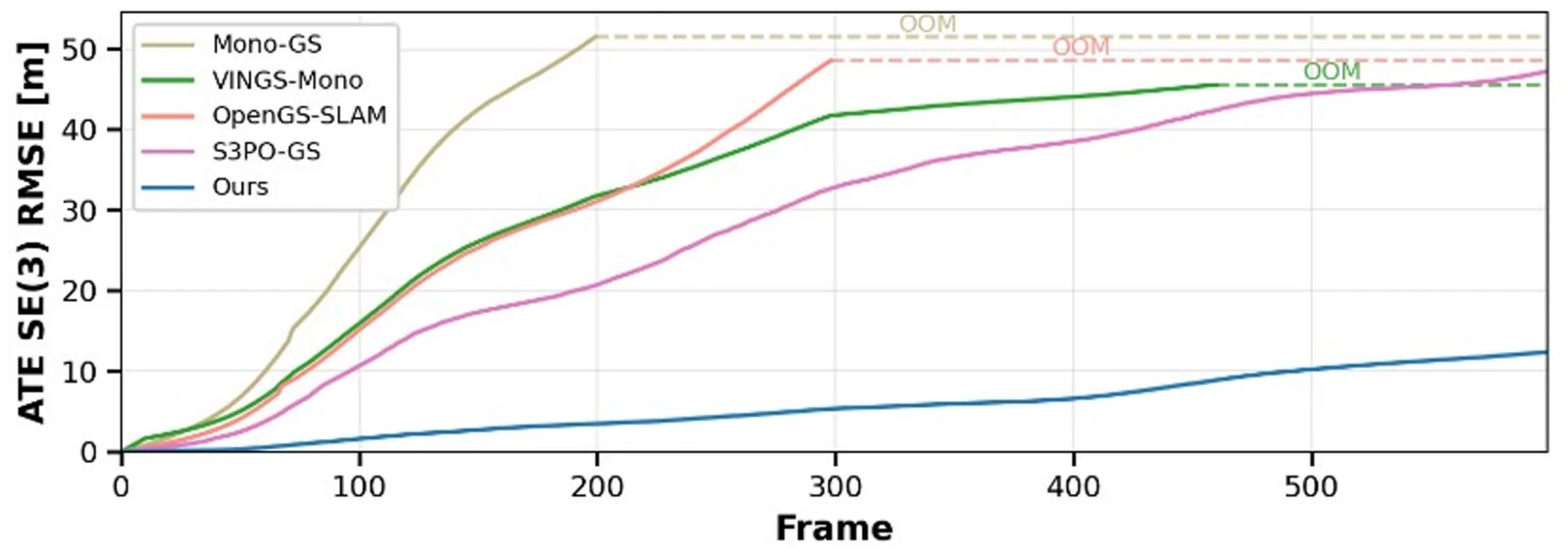
Extended stress-test ATE-SE(3) RMSE versus frame index on a single KITTI Odometry sequence (07, first 600 frames) under a fixed GPU memory budget. This panel is a controlled length stress check for the reported setting, not broad evidence of long-horizon scalability.

As a single-sequence extended stress test under a fixed GPU memory budget—not as broad evidence of long-horizon or large-scale scalability—we run the first 600 frames of KITTI 07. As shown in Figure 11, most baselines exhibit early out-of-memory (OOM) while their ATE-SE(3) already exceeds 45 m; S3PO-GS completes 600 frames but with large drift, whereas MoGe2-SLAM proceeds farther with lower drift than these compared baselines under the reported setting. Because this panel uses only one extended sequence, we treat it as a controlled stress check that motivates the fixed-budget reachability analysis.

## 5. Limitations

Table 11’s failure modes persist in settings the suite does not fully cover. Weak-parallax highway motion remains the main accuracy failure (KITTI 01 ATE-SE(3) = 8.42 m; local error ≈ 20 m), and one occlusion window is degenerate (Argoverse 2 30e94a6b; Fail). On extended KITTI prefixes, ATE-SE(3) also grows with sequence length (sequence 07 reaches 6.67 m at 600 frames), alongside memory and map growth. The dynamic-traffic and illumination clips in Table 11 finish at 0.29/0.34 m and 1.65/1.35 m, but these windows are still far from the harder regimes of severe object dominance and extreme low light, which therefore remain open. Runtime remains a constraint. Mean tracking throughput is about 1.04 Hz on KITTI 07 (Table 12), so the system is intended for offline or near-online evaluation rather than real-time (10 Hz) operation. Future work will investigate weak-parallax constraints, occlusion-aware mapping, dynamic-scene filtering, robustness under extreme low light, and lightweight large-scale map maintenance.

## 6. Conclusions

This paper presents MoGe2-SLAM, a monocular 3DGS SLAM system for outdoor driving that recovers approximate the absolute metric scale from a single RGB stream and known camera height only. The method uses the MoGe-2 depth prior together with ground-plane geometry and camera height to complete Absolute-Scale Initialization from the first keyframe; a Grid-Balanced Tracking front end and ORB-Anchored Mapping back end jointly propagate scale information and optimize 3D Gaussians so that the approximate absolute metric scale and high-fidelity appearance co-evolve. Most importantly, the resulting metrically and multi-view consistent Gaussian map can provide high-quality structured geometric priors for downstream tasks requiring geometric consistency such as world models.

## Figures and Tables

**Table 1:** Tracking performance comparison on KITTI. **Bold** entries denote the best method, and underlined entries denote the second-best method.

Method	Category	ATE-Sim(3) ↓	ATE-SE(3) ↓
ORB-SLAM3 [19]	point map/VO	0.890	38.963
VGGT-Long [56]		1.866	41.376
ORB-SLAM2-HScale [46]		13.18	17.31
LIBVISO2 Mono [45]		14.55	24.85
NeRF-SLAM [33]	NeRF	16.458	17.456
NICER-SLAM [34]		18.531	23.957
MonoGS [36]	3DGS	6.259	51.601
VINGS-Mono [41]		1.024	37.242
OpenGS-SLAM [42]		4.418	51.762
S3PO-GS [43]		1.106	25.129
**MoGe2-SLAM (Ours)**		**0.747 ± 0.356**	**2.583 ± 0.631**

**Table 2:** Tracking performance comparison on Waymo. **Bold** entries denote the best method, and underlined entries denote the second-best method.

Method	Category	ATE-Sim(3) ↓	ATE-SE(3) ↓
ORB-SLAM3 [19]	point map/VO	**0.419**	33.060
VGGT-Long [56]		1.471	36.180
ORB-SLAM2-HScale [46]		2.597	**5.134**
LIBVISO2 Mono [45]		1.518	5.468
NeRF-SLAM [33]	NeRF	OOM	OOM
NICER-SLAM [34]		15.881	19.115
MonoGS [36]	3DGS	8.530	53.235
VINGS-Mono [41]		3.130	68.406
OpenGS-SLAM [42]		0.839	36.023
S3PO-GS [43]		0.622	26.648
**MoGe2-SLAM (Ours)**		0.693 ± 0.135	5.463 ± 0.327

**Table 3:** Tracking performance comparison on Argoverse 2. **Bold** entries denote the best method, and underlined entries denote the second-best method.

Method	Category	ATE-Sim(3) ↓	ATE-SE(3) ↓
ORB-SLAM3 [19]	point map/VO	0.582	34.820
VGGT-Long [56]		1.640	38.215
ORB-SLAM2-HScale [46]		1.415	6.439
LIBVISO2 Mono [45]		2.436	22.724
NeRF-SLAM [33]	NeRF	14.820	16.905
NICER-SLAM [34]		16.440	21.330
MonoGS [36]	3DGS	3.728	47.812
VINGS-Mono [41]		1.227	39.441
OpenGS-SLAM [42]		2.240	44.180
S3PO-GS [43]		0.581	21.342
**MoGe2-SLAM (Ours)**		**0.510 ± 0.163**	**3.920 ± 0.889**

**Table 4:** Dataset-level metric-scale diagnostics for MoGe2-SLAM. ρ is the path-length ratio before SE(3) alignment; *ε*_s_ = |ρ − 1|; ρ_(k)_ is the path-length ratio on the k-th 50 m segment (ΔL = 50 m). RPE_t_/RPE_r_ are means of per-clip translational/rotational RPE.

Dataset	Mean ρ (%)	Mean ε_s_	RPE_t_ (m)	RPE_r_ (°)	Mean Max |ρ_(k)_ − 1|
KITTI	93.37	0.066	0.21	0.15	0.090
Waymo	83.42	0.166	0.45	0.17	0.172
Argoverse 2	93.72	0.065	2.67	2.06	0.071
average	90.17	0.103	0.91	0.63	0.116

**Table 5:** Mapping performance comparison on KITTI. **Bold** entries denote the best method, and underlined entries denote the second-best method.

Method	PSNR ↑	SSIM ↑	LPIPS ↓
NeRF-SLAM [33]	11.33	0.44	0.77
NICER-SLAM [34]	12.69	0.44	0.68
MonoGS [36]	14.10	0.46	0.75
VINGS-Mono [41]	16.14	0.56	0.57
OpenGS-SLAM [42]	14.54	0.51	0.66
S3PO-GS [43]	20.01	0.64	0.41
**MoGe2-SLAM (Ours)**	**21.29 ± 0.15**	**0.70 ± 0.006**	**0.30 ± 0.005**

**Table 6:** Mapping performance comparison on Waymo. **Bold** entries denote the best method, and underlined entries denote the second-best method.

Method	PSNR ↑	SSIM ↑	LPIPS ↓
NeRF-SLAM [33]	OOM	OOM	OOM
NICER-SLAM [34]	12.32	0.62	0.72
MonoGS [36]	21.80	0.78	0.58
VINGS-Mono [41]	20.66	0.69	0.51
OpenGS-SLAM [42]	23.99	0.80	0.43
S3PO-GS [43]	**26.72**	0.84	0.36
**MoGe2-SLAM (Ours)**	26.68 ± 0.26	**0.85 ± 0.003**	**0.31 ± 0.004**

**Table 7:** Mapping performance comparison on Argoverse 2. **Bold** entries denote the best method, and underlined entries denote the second-best method.

Method	PSNR ↑	SSIM ↑	LPIPS ↓
NeRF-SLAM [33]	12.10	0.42	0.73
NICER-SLAM [34]	13.05	0.48	0.69
MonoGS [36]	19.59	0.64	0.74
VINGS-Mono [41]	15.20	0.49	0.65
OpenGS-SLAM [42]	20.50	0.68	0.71
S3PO-GS [43]	25.83	**0.76**	0.49
**MoGe2-SLAM (Ours)**	**25.87 ± 0.14**	**0.76 ± 0.004**	**0.45 ± 0.003**

**Table 8:** Module ablation on nine KITTI sequences (means). MASt3R w/ camera height: MASt3R prior with *h_c_* kept. MASt3R w/o camera height: MASt3R prior with *h_c_* off. MoGe-2 w/o camera height: MoGe-2 prior with the native scale head still run and *h_c_* off.

Variant	ATE-Sim(3) ↓	ATE-SE(3) ↓	PSNR ↑	SSIM ↑	LPIPS ↓
MASt3R w/ camera height	0.984	16.952	20.82	0.66	0.35
MASt3R w/o camera height	0.968	15.104	20.90	0.68	0.33
MoGe-2 w/o camera height	1.103	5.944	20.91	0.66	0.33
w/o Grid-Balanced Tracking	1.392	3.864	20.94	0.67	0.32
w/o ORB-Anchored Mapping	1.253	4.706	20.41	0.63	0.38
MoGe2-SLAM (Ours)	0.747	2.583	21.29	0.70	0.30

**Table 9:** Controlled Grid-Balanced Tracking ablation on the weak-texture window of KITTI 03. Inlier: mean RANSAC-PnP inlier ratio. Cov.: occupancy-grid coverage (fraction of grid cells that contain at least one selected correspondence). #Corr.: mean correspondence count. Full Grid-Balanced Tracking is G = 8 with CLAHE and edge enhancement.

Variant	Inlier ↑	Cov. ↑	#Corr.	ATE-Sim(3) ↓	ATE-SE(3) ↓
Conventional ORB	0.898	0.301	163.4	2.178	10.611
Uniform grid	0.862	0.374	114.7	2.106	9.992
Grid 6 × 6	0.867	0.461	95.0	2.331	8.637
Grid 10 × 10	0.878	0.339	196.9	2.385	10.273
Grid 12 × 12	0.883	0.303	251.9	1.973	8.816
Grid 16 × 16	0.877	0.246	360.8	2.327	9.607
w/o CLAHE	0.837	0.405	168.9	4.216	10.600
w/o edge enhancement	0.869	0.409	199.0	2.443	9.735
Full Grid-Balanced Tracking (Ours)	0.869	0.393	142.9	1.885	8.695

**Table 10:** Camera-height and ground-plane sensitivity. ATE-SE(3) is short-clip keyframe root-mean-square error (RMSE) (m); ρ is the path-length ratio before SE(3) alignment and *ε*_s_ = |ρ − 1|. Incorrect *h_c_* is the mean over KITTI 00/05/07. All other rows are KITTI 07; ρ and *ε*_s_ are recomputed from the same saved trajectories underlying the ATE values.

Stressor	Setting	ATE SE(3) (m)	ρ	ε_s_
Incorrect h_c_	δh_c_ = −15%	0.55	0.861	0.139
Incorrect h_c_	δh_c_ = −10%	0.81	0.856	0.144
Incorrect h_c_	δh_c_ = −5%	0.53	0.877	0.123
Incorrect h_c_	δh_c_ = 0% (nominal)	0.62	0.873	0.127
Incorrect h_c_	δh_c_ = +5%	0.63	0.876	0.124
Incorrect h_c_	δh_c_ = +10%	0.53	0.881	0.119
Incorrect h_c_	δh_c_ = +15%	0.66	0.877	0.123
Road tilt	±3°/±5°/±8°	0.34	0.913	0.087
Pitch	+5° (worst)	0.54	0.923	0.077
Pitch	±2° mean	0.34	0.913	0.087
Roll	−5° (worst)	0.46	0.918	0.082
Roll	±2° mean	0.33	0.912	0.088
Uneven ground	σ = 0.02–0.10 m	0.36	0.910	0.090
Ground occlusion	ω = 25% ROI	0.33	0.911	0.089
Ground occlusion	ω = 50% ROI	0.51	0.922	0.078
Ground occlusion	ω = 75% ROI	0.43	0.916	0.084

**Table 11:** Hard-case suite. ATE-SE(3) is keyframe root-mean-square error (RMSE) (m), reported separately for Ours and S3PO-GS. Fail marks a lost track or a degenerate window that is not counted as a successful metric result (both methods: Argoverse 2 30e94a6b).

Stressor	Sequence	Frames	MoGe2-SLAM (Ours) ATE-SE(3) (m)	S3PO-GS [43] ATE-SE(3) (m)
Dynamic traffic	Argoverse 2 48c9cd36	200–318	0.29	0.21
Dynamic traffic	Waymo 13476	0–196	0.34	1.61
Weak parallax	KITTI 01	0–199	8.42	14.92
Weak parallax	KITTI 07	400–599	1.47	4.20
Occlusion	Argoverse 2 30e94a6b	200–318	Fail	Fail
Occlusion	Argoverse 2 4612f4a4	200–315	0.46	0.17
Illumination	Waymo 153495	0–198	1.65	2.17
Illumination	KITTI 07	200–399	1.35	4.01

**Table 12:** KITTI runtime profiling on NVIDIA RTX A6000 (mean values). Trk: mean per-frame tracking time; Map: mean keyframe mapping time; Peak: peak GPU memory; |G|: active Gaussian count (×10^5^). Approximate tracking throughput ≈ 1/Trk.

Method	Trk (s) ↓	Map (s) ↓	Peak (GB) ↓	|G| (×10^5^)
MonoGS [36]	0.643	1.053	9.0	1.806
S3PO-GS [43]	1.028	1.922	9.5	1.955
MoGe2-SLAM (Ours)	0.961	2.030	8.2	1.768

## Data Availability

The original data presented in the study are openly available in the KITTI Odometry benchmark at https://www.cvlibs.net/datasets/kitti/ (accessed on 21 November 2025) (reference [61]), the Waymo Open Dataset at https://waymo.com/open/ (accessed on 28 October 2025) (reference [62]), and Argoverse 2 at https://www.argoverse.org/av2.html (accessed on 5 November 2025) (reference [63]).

## Supplementary Materials

The following supporting information can be downloaded at: https://www.mdpi.com/article/10.3390/s26206388/s1, Figure S1: Scale error versus frame index; Table S1: Exact evaluation sequences used in this work (nine clips per dataset); Table S2: Per-sequence ATE on KITTI Odometry; Table S3: Per-sequence novel-view metrics on KITTI Odometry; Table S4: Per-sequence ATE on Waymo Open Dataset; Table S5: Per-sequence novel-view metrics on Waymo Open Dataset; Table S6: Per-sequence ATE on Argoverse 2; Table S7: Per-sequence novel-view metrics on Argoverse 2; Table S8: Per-sequence metric-scale diagnostics for MoGe2-SLAM; Table S9: Depth-prior swap on KITTI 00, 05, and 07; Table S10: Module ablation on nine Waymo FRONT clips; Table S11: Module ablation on nine Argoverse 2 clips; Table S12: Key hyperparameters used in all reported experiments; Table S13: Prefix ATE-SE(3) and resource traces on extended KITTI logs.
