# Supplementary material for: MoGe2-SLAM: Outdoor Monocular 3D Gaussian Splatting SLAM with Absolute Scale
Source: Sensors (Basel). 2026 Oct 9;26(20):6388. doi: 10.3390/s26206388 (PMC13709284; doi:10.3390/s26206388)
Supplement: Supplementary file 1 [file sensors-26-06388-s001.zip › sensors-4553759-supplementary.pdf]

Supplementary Materials

Manuscript ID: sensors-4553759 — MoGe2-SLAM: Outdoor Monocular 3D Gaussian Splatting SLAM with Absolute Scale

This file contains Tables S1–S13 and Figure S1. Dataset-level means, the nine-KITTI module ablation (Table 8), the Grid-Balanced Tracking sheet (Table 9), camera-height sensitivity (Table 10), and the hard-case suite (Table 11) remain in the main text. Tables S10 and S11 repeat Table 8's variants as nine-clip means on Waymo and Argoverse 2. Tables S9–S13 are additional revision sheets and are not merged into main-text Tables 1–7.

S1. Evaluation protocol (sequence IDs, counts, frame ranges, and selection criteria)

Number of sequences. The main quantitative study uses 27 outdoor clips in total: nine sequences from each of KITTI Odometry, Waymo Open Dataset, and Argoverse 2. Unless otherwise stated, dataset-level ATE / novel-view means in Section 4 of the main text are averages over the nine clips of that dataset (27 clips overall).

Clip length / frame ranges. KITTI clips are contiguous 200-frame windows (inclusive start–end indices in Table S0), following the S3PO-GS outdoor protocol. Waymo clips use the prepared FRONT/rgb image streams for the same S3PO-GS sequence IDs; the available lengths on disk are 197–199 frames (exact inclusive indices and counts in Table S0). Argoverse 2 clips use the first 200 frames of each selected log (indices 0–199).

Selection criteria. (i) KITTI and Waymo: we follow the outdoor monocular 3DGS evaluation sequence IDs used by S3PO-GS (not a MoGe2-SLAM-specific shortlist). (ii) Argoverse 2: we select nine outdoor urban logs and take the first 200 frames of each; these clips still contain residual traffic, occlusion, and illumination change. Across all three datasets, clips are outdoor drives with sufficient camera baseline / viewpoint change for monocular geometry to be well posed. Section 4.6 of the main text reports a hard-case suite and tracking failures.

Table S1. Exact evaluation sequences used in this work (nine clips per dataset). Waymo Length equals the number of FRONT/rgb frames available for that sequence. This sheet supports the Section 4.1 protocol summary in the main text (the main text no longer reprints a 27-row sequence table).

Dataset	Sequence ID	Frame range (inclusive)	Length
KITTI Odometry	00	480–679	200
KITTI Odometry	02	1490–1689	200
KITTI Odometry	03	280–479	200
KITTI Odometry	05	0–199	200
KITTI Odometry	06	160–359	200
KITTI Odometry	07	0–199	200
KITTI Odometry	08	2128–2327	200
KITTI Odometry	09	0–199	200
KITTI Odometry	10	89–288	200
Waymo Open Dataset	100613	0–197	198
Waymo Open Dataset	106762	0–197	198
Waymo Open Dataset	132384	0–198	199
Waymo Open Dataset	13476	0–196	197
Waymo Open Dataset	152706	0–198	199
Waymo Open Dataset	153495	0–198	199
Waymo Open Dataset	158686	0–197	198
Waymo Open Dataset	163453	0–197	198
Waymo Open Dataset	405841	0–198	199
Argoverse 2	1ad57a00	0–199	200
Argoverse 2	1ca5291b	0–199	200
Argoverse 2	14896a70	0–199	200
Argoverse 2	2a930061	0–199	200
Argoverse 2	30e94a6b	0–199	200

Argoverse 2	3503b283	0–199	200
Argoverse 2	4fae2ef6	0–199	200
Argoverse 2	4612f4a4	0–199	200
Argoverse 2	48c9cd36	0–199	200

Tables S2–S7 below expand the main-text dataset-level means to per-sequence scores, using the same method ordering as Section 4.

ATE tables report ATE-Sim(3) and ATE-SE(3) (meters). NVS tables report PSNR / SSIM / LPIPS. Empty cells marked "—" are not available at the per-sequence level. "OOM" follows the main-text notation for out-of-memory failures. In each numeric column the best score is shaded blue and the second-best orange.

Table S2. Per-sequence ATE on KITTI Odometry (corresponds to the main-text KITTI ATE table).

Method	Metric	00	02	03	05	06	07	08	09	10	Avg
ORB-SLAM3	ATE-Sim(3) ↓	0.753	0.735	0.217	1.077	3.763	0.736	0.468	0.139	0.126	**0.890**
	ATE-SE(3) ↓	27.472	58.732	43.979	37.084	29.154	29.434	28.910	54.517	41.386	**38.963**
VGGT-Long	ATE-Sim(3) ↓	0.790	1.572	1.983	2.104	3.587	1.083	0.896	2.662	2.121	**1.866**
	ATE-SE(3) ↓	28.436	63.499	47.887	39.411	30.682	31.095	30.580	56.424	44.372	**41.376**
ORB-SLAM2-HScale	ATE-Sim(3) ↓	18.992	21.027	10.357	0.769	13.657	0.672	43.407	6.423	3.279	**13.18**
	ATE-SE(3) ↓	19.476	33.143	14.619	1.616	21.301	0.689	51.780	6.728	6.441	**17.31**
LIBVISO2 Mono	ATE-Sim(3) ↓	17.13	20.28	10.09	2.15	18.60	1.99	40.19	8.14	12.36	**14.55**
	ATE-SE(3) ↓	17.73	20.49	12.03	9.07	19.45	3.49	50.62	13.73	77.10	**24.85**
NeRF-SLAM	ATE-Sim(3) ↓	17.101	20.3	13.811	10.597	45.336	1.854	20.112	12.02	6.991	**16.458**
	ATE-SE(3) ↓	17.9	20.866	14.561	11.203	47.239	1.95	21.6	13.777	7.009	**17.456**
NICER-SLAM	ATE-Sim(3) ↓	6.885	16.213	22.998	25.097	30.121	1.006	20.116	33.969	10.374	**18.531**
	ATE-SE(3) ↓	7.01	18.745	24.325	26.04	33.726	1.944	49.523	42.1	12.2	**23.957**
MonoGS	ATE-Sim(3) ↓	8.889	9.114	5.949	3.407	3.272	6.097	5.829	6.934	6.839	**6.259**
	ATE-SE(3) ↓	50.227	56.396	48.675	40.118	46.971	63.02	69.433	40.011	40.558	**51.601**
VINGS-Mono	ATE-Sim(3) ↓	1.129	0.333	0.429	1.342	3.362	1.412	0.198	0.615	0.400	**1.024**
	ATE-SE(3) ↓	28.764	12.345	33.21	45.678	51.234	40.133	19.866	62.344	41.605	**37.242**
OpenGS-SLAM	ATE-Sim(3) ↓	1.027	5.193	3.096	3.015	12.127	3.438	0.737	4.132	7.000	**4.418**
	ATE-SE(3) ↓	23.891	67.210	45.331	39.991	82.33	56.708	18.765	57.76	74.139	**51.762**
S3PO-GS	ATE-Sim(3) ↓	0.315	1.137	2.617	0.880	0.473	0.557	1.342	1.568	1.068	**1.106**
	ATE-SE(3) ↓	24.256	27.418	48.293	16.309	3.847	9.624	42.087	19.756	34.571	**25.129**
**MoGe2-SLAM (Ours)**	ATE-Sim(3) ↓	0.302	0.490	0.450	0.749	1.330	0.647	0.797	0.912	1.050	**0.747**
	ATE-SE(3) ↓	0.9561	4.020	5.3985	1.000	1.830	0.6715	0.8098	2.0645	6.4942	**2.583**

Table S3. Per-sequence novel-view metrics on KITTI Odometry (corresponds to the main-text KITTI NVS table).

Method	Metric	00	02	03	05	06	07	08	09	10	Avg
NeRF-SLAM	PSNR ↑	11.345	9.322	11.351	12.308	11.337	11.316	11.329	12.312	10.35	**11.33**
	SSIM ↑	0.428	0.551	0.439	0.342	0.437	0.443	0.440	0.479	0.401	**0.44**
	LPIPS ↓	0.768	0.771	0.769	0.772	0.767	0.773	0.770	0.769	0.771	**0.77**
NICER-SLAM	PSNR ↑	12.688	13.691	13.689	11.692	12.687	12.693	12.690	13.689	10.691	**12.69**
	SSIM ↑	0.438	0.541	0.339	0.442	0.437	0.443	0.440	0.339	0.541	**0.44**
	LPIPS ↓	0.678	0.681	0.679	0.682	0.677	0.683	0.680	0.679	0.681	**0.68**

MonoGS	PSNR ↑	15.832	9.748	16.777	16.125	16.197	11.254	15.416	13.545	11.960	**14.09**
	SSIM ↑	0.579	0.213	0.520	0.547	0.579	0.360	0.542	0.477	0.339	**0.46**
	LPIPS ↓	0.691	0.800	0.726	0.697	0.715	0.783	0.741	0.776	0.794	**0.75**
VINGS-Mono	PSNR ↑	16.834	17.445	17.916	16.629	16.089	15.026	16.834	15.144	13.372	**16.14**
	SSIM ↑	0.598	0.550	0.552	0.576	0.545	0.586	0.599	0.522	0.474	**0.56**
	LPIPS ↓	0.517	0.592	0.581	0.546	0.575	0.529	0.522	0.615	0.632	**0.57**
OpenGS-SLAM	PSNR ↑	15.452	14.112	15.773	15.970	13.938	14.127	15.795	13.258	12.455	**14.54**
	SSIM ↑	0.567	0.470	0.491	0.541	0.531	0.517	0.548	0.463	0.448	**0.51**
	LPIPS ↓	0.578	0.680	0.649	0.638	0.700	0.655	0.592	0.711	0.705	**0.66**
S3PO-GS	PSNR ↑	21.160	19.390	20.260	20.740	20.720	20.080	20.330	19.850	17.580	**20.01**
	SSIM ↑	0.740	0.590	0.574	0.662	0.656	0.706	0.689	0.587	0.550	**0.64**
	LPIPS ↓	0.265	0.442	0.483	0.379	0.397	0.349	0.324	0.547	0.545	**0.41**
**MoGe2-SLAM (Ours)**	PSNR ↑	22.618	21.170	21.770	23.333	20.367	22.149	21.784	20.287	18.129	**21.29**
	SSIM ↑	0.787	0.672	0.659	0.766	0.673	0.762	0.753	0.655	0.594	**0.70**
	LPIPS ↓	0.184	0.326	0.337	0.236	0.344	0.243	0.243	0.323	0.426	**0.30**

Table S4. Per-sequence ATE on Waymo Open Dataset (corresponds to the main-text Waymo ATE table).

**Method**	**Metric**	**13476**	**100613**	**106762**	**132384**	**152706**	**158686**	**153495**	**163453**	**405841**	**Avg**
ORB-SLAM3	ATE-Sim(3) ↓	0.466	0.256	0.129	0.057	0.310	0.246	0.476	0.518	1.312	**0.419**
	ATE-SE(3) ↓	15.497	30.287	59.636	51.743	35.450	20.832	43.908	19.949	20.235	**33.060**
VGGT-Long	ATE-Sim(3) ↓	1.323	2.638	1.672	0.703	0.620	1.176	1.577	1.916	1.616	**1.471**
	ATE-SE(3) ↓	33.909	61.349	55.256	16.523	36.745	29.991	24.128	45.012	22.704	**36.180**
ORB-SLAM2-HScale	ATE-Sim(3) ↓	1.78	1.019	0.831	14.008	1.758	0.663	1.57	1.257	0.486	**2.597**
	ATE-SE(3) ↓	3.827	2.239	2.701	23.816	2.590	2.456	6.186	1.909	0.487	**5.134**
LIBVISO2 Mono	ATE-Sim(3) ↓	2.09	0.63	0.75	1.45	0.46	1.88	1.55	3.74	1.11	**1.518**
	ATE-SE(3) ↓	3.70	3.89	0.80	2.80	3.30	3.59	5.49	19.26	6.38	**5.468**
NeRF-SLAM	ATE-Sim(3) ↓	OOM	OOM	OOM	OOM	OOM	OOM	OOM	OOM	OOM	**OOM**
	ATE-SE(3) ↓	OOM	OOM	OOM	OOM	OOM	OOM	OOM	OOM	OOM	**OOM**
NICER-SLAM	ATE-Sim(3) ↓	5.137	23.864	8.492	37.215	12.609	19.358	2.741	27.483	6.030	**15.881**
	ATE-SE(3) ↓	7.294	24.508	10.873	39.912	13.627	25.341	14.056	28.479	7.945	**19.115**
MonoGS	ATE-Sim(3) ↓	3.366	6.953	18.160	12.080	9.180	8.396	5.718	11.210	1.703	**8.530**
	ATE-SE(3) ↓	18.473	92.615	34.208	127.539	7.842	68.914	51.327	44.086	34.111	**53.235**
VINGS-Mono	ATE-Sim(3) ↓	2.243	1.617	5.260	3.735	2.200	3.687	3.615	3.524	2.288	**3.130**
	ATE-SE(3) ↓	9.573	132.846	48.291	95.407	27.618	81.752	63.019	72.384	84.764	**68.406**
OpenGS-SLAM	ATE-Sim(3) ↓	0.422	0.324	0.893	0.436	0.309	1.076	1.576	1.719	0.800	**0.839**
	ATE-SE(3) ↓	4.916	82.537	27.184	53.692	11.408	61.275	38.946	19.723	24.526	**36.023**
S3PO-GS	ATE-Sim(3) ↓	0.498	0.476	0.270	0.520	0.347	0.830	1.200	0.972	0.484	**0.622**
	ATE-SE(3) ↓	2.847	64.391	15.726	39.508	8.163	52.974	21.385	16.942	17.896	**26.648**
**MoGe2-SLAM (Ours)**	ATE-Sim(3) ↓	0.502	0.399	0.752	0.920	0.330	0.509	1.350	0.806	0.668	**0.693**
	ATE-SE(3) ↓	0.9336	3.1678	7.0184	15.155	4.2116	4.0032	3.9693	8.4128	2.2932	**5.463**

Table S5. Per-sequence novel-view metrics on Waymo Open Dataset (corresponds to the main-text Waymo NVS table).

Method	Metric	13476	100613	106762	132384	152706	158686	153495	163453	405841	Avg
NeRF-SLAM	PSNR ↑	OOM	OOM	OOM	OOM	OOM	OOM	OOM	OOM	OOM	**OOM**
	SSIM ↑	OOM	OOM	OOM	OOM	OOM	OOM	OOM	OOM	OOM	**OOM**
	LPIPS ↓	OOM	OOM	OOM	OOM	OOM	OOM	OOM	OOM	OOM	**OOM**
NICER-SLAM	PSNR ↑	12.144	12.636	12.908	13.077	12.554	11.061	11.87	11.289	13.341	**12.32**
	SSIM ↑	0.617	0.624	0.599	0.643	0.618	0.621	0.620	0.616	0.622	**0.62**
	LPIPS ↓	0.717	0.724	0.719	0.723	0.718	0.821	0.720	0.716	0.622	**0.72**
MonoGS	PSNR ↑	20.950	21.890	22.240	23.480	22.520	21.250	21.490	19.280	23.140	**21.80**
	SSIM ↑	0.723	0.779	0.814	0.856	0.791	0.734	0.782	0.743	0.804	**0.78**
	LPIPS ↓	0.693	0.543	0.515	0.427	0.649	0.574	0.635	0.642	0.522	**0.58**
VINGS-Mono	PSNR ↑	16.150	22.696	23.117	23.774	19.264	21.428	20.283	16.880	22.381	**20.66**
	SSIM ↑	0.612	0.731	0.748	0.803	0.672	0.674	0.673	0.578	0.732	**0.69**
	LPIPS ↓	0.578	0.474	0.436	0.364	0.585	0.503	0.553	0.641	0.462	**0.51**
OpenGS-SLAM	PSNR ↑	22.290	24.410	26.190	26.980	23.950	21.710	23.660	21.000	25.720	**23.99**
	SSIM ↑	0.733	0.811	0.851	0.883	0.802	0.731	0.800	0.745	0.840	**0.80**
	LPIPS ↓	0.602	0.360	0.326	0.283	0.533	0.468	0.499	0.506	0.333	**0.43**
S3PO-GS	PSNR ↑	25.924	25.567	29.284	26.620	28.974	25.860	26.802	24.271	27.208	**26.72**
	SSIM ↑	0.797	0.829	0.889	0.880	0.864	0.817	0.853	0.808	0.863	**0.84**
	LPIPS ↓	0.445	0.313	0.274	0.276	0.416	0.376	0.373	0.397	0.352	**0.36**
**MoGe2-SLAM (Ours)**	PSNR ↑	26.271	27.717	26.812	25.606	29.705	27.171	25.739	23.337	27.799	**26.68**
	SSIM ↑	0.808	0.863	0.853	0.868	0.873	0.849	0.838	0.797	0.871	**0.85**
	LPIPS ↓	0.380	0.279	0.282	0.278	0.342	0.277	0.349	0.336	0.266	**0.31**

Table S6. Per-sequence ATE on Argoverse 2 (corresponds to the main-text Argoverse 2 ATE table).

Method	Metric	1ad57a00	1ca5291b	14896a70	2a930061	30e94a6b	3503b283	4fae2ef6	4612f4a4	48c9cd36	Avg
ORB-SLAM3	ATE-Sim(3) ↓	0.634	1.320	0.912	0.443	0.756	0.198	0.301	0.587	0.087	**0.582**
	ATE-SE(3) ↓	35.642	77.011	48.213	27.543	41.876	18.234	22.876	32.109	9.876	**34.82**
VGGT-Long	ATE-Sim(3) ↓	1.654	4.320	2.312	1.201	1.987	0.654	0.987	1.432	0.213	**1.640**
	ATE-SE(3) ↓	39.012	83.633	52.134	30.123	45.678	19.876	25.456	35.678	12.345	**38.215**
ORB-SLAM2-HScale	ATE-Sim(3) ↓	0.936	0.013	1.800	0.165	5.929	0.192	0.381	2.128	1.187	**1.415**
	ATE-SE(3) ↓	5.779	0.152	9.487	4.854	12.675	0.432	4.441	10.492	9.637	**6.439**
LIBVISO2 Mono	ATE-Sim(3) ↓	1.275	0.07	3.812	3.36	2.79	2.17	2.32	3.43	2.70	**2.436**
	ATE-SE(3) ↓	21.483	36.96	30.096	48.34	12.56	4.13	19.92	18.77	12.26	**22.724**
NeRF-SLAM	ATE-Sim(3) ↓	14.907	34.472	21.718	11.492	18.453	6.284	9.371	13.646	3.037	**14.820**
	ATE-SE(3) ↓	16.782	39.817	25.354	12.636	20.609	7.491	10.048	15.273	4.135	**16.905**
NICER-SLAM	ATE-Sim(3) ↓	16.574	39.327	24.518	12.287	20.446	7.312	9.645	14.393	3.458	**16.440**
	ATE-SE(3) ↓	21.292	50.889	31.147	15.328	26.234	9.374	12.916	18.563	6.227	**21.330**
MonoGS	ATE-Sim(3) ↓	1.844	6.347	4.927	3.890	3.950	1.177	1.461	8.979	0.977	**3.728**
	ATE-SE(3) ↓	49.412	101.041	65.032	38.901	56.779	25.648	32.385	45.628	15.482	**47.812**
VINGS-Mono	ATE-Sim(3) ↓	2.369	6.802	0.026	0.781	0.051	0.201	0.650	0.120	0.047	**1.227**
	ATE-SE(3) ↓	40.446	82.857	53.789	32.355	47.143	21.214	26.709	37.930	12.527	**39.441**
OpenGS-SLAM	ATE-Sim(3) ↓	2.171	3.776	3.931	2.897	0.682	1.412	0.664	2.049	2.581	**2.240**
	ATE-SE(3) ↓	45.789	90.538	60.123	36.789	52.456	24.567	30.123	42.345	14.890	**44.18**
S3PO-GS	ATE-Sim(3) ↓	0.311	0.348	0.265	0.265	0.854	0.803	0.445	0.987	0.949	**0.581**
	ATE-SE(3) ↓	22.335	52.238	29.062	16.543	25.648	9.072	12.779	19.886	4.517	**21.342**

MoGe2-SLAM (Ours)	ATE-Sim(3) ↓	0.471	0.715	1.425	0.331	0.500	0.270	0.320	0.353	0.205	**0.510**
	ATE-SE(3) ↓	4.173	9.864	5.678	2.850	4.901	1.496	2.163	3.527	0.628	**3.920**

Table S7. Per-sequence novel-view metrics on Argoverse 2 (corresponds to the main-text Argoverse 2 NVS table).

Method	Metric	1ad57a00	1ca5291b	14896a70	2a930061	30e94a6b	3503b283	4fae2ef6	4612f4a4	48c9cd36	Avg
NeRF-SLAM	PSNR ↑	13.093	10.104	12.087	12.112	12.098	12.106	12.081	12.119	13.100	**12.10**
	SSIM ↑	0.413	0.428	0.405	0.437	0.419	0.422	0.408	0.431	0.417	**0.42**
	LPIPS ↓	0.724	0.738	0.715	0.746	0.729	0.732	0.718	0.741	0.727	**0.73**
NICER-SLAM	PSNR ↑	14.042	10.058	13.031	14.067	13.048	14.053	13.037	13.064	13.050	**13.05**
	SSIM ↑	0.473	0.489	0.465	0.497	0.479	0.482	0.468	0.491	0.476	**0.48**
	LPIPS ↓	0.683	0.699	0.675	0.706	0.687	0.692	0.678	0.701	0.689	**0.69**
MonoGS	PSNR ↑	22.347	10.310	20.791	20.581	21.050	21.305	20.223	19.250	20.456	**19.59**
	SSIM ↑	0.724	0.240	0.741	0.686	0.729	0.718	0.674	0.644	0.620	**0.64**
	LPIPS ↓	0.676	0.905	0.697	0.730	0.687	0.728	0.714	0.755	0.780	**0.74**
VINGS-Mono	PSNR ↑	11.289	9.175	16.268	13.071	13.390	20.904	14.081	19.566	19.055	**15.20**
	SSIM ↑	0.316	0.237	0.503	0.456	0.585	0.654	0.551	0.541	0.560	**0.49**
	LPIPS ↓	0.771	0.787	0.663	0.674	0.564	0.523	0.623	0.608	0.622	**0.65**
OpenGS-SLAM	PSNR ↑	22.766	20.152	19.947	20.631	22.944	21.538	17.519	19.204	19.755	**20.50**
	SSIM ↑	0.725	0.653	0.729	0.683	0.741	0.718	0.625	0.643	0.614	**0.68**
	LPIPS ↓	0.670	0.716	0.710	0.712	0.643	0.729	0.743	0.734	0.769	**0.71**
S3PO-GS	PSNR ↑	26.744	25.137	27.475	27.549	24.900	27.432	25.113	23.534	24.630	**25.83**
	SSIM ↑	0.785	0.716	0.817	0.788	0.772	0.786	0.756	0.694	0.690	**0.76**
	LPIPS ↓	0.422	0.523	0.463	0.418	0.488	0.505	0.465	0.584	0.518	**0.49**
**MoGe2-SLAM (Ours)**	PSNR ↑	25.517	25.887	24.447	26.260	25.335	25.750	25.947	26.524	27.143	**25.87**
	SSIM ↑	0.786	0.730	0.763	0.758	0.778	0.763	0.777	0.713	0.759	**0.76**
	LPIPS ↓	0.411	0.481	0.460	0.416	0.518	0.467	0.387	0.487	0.399	**0.45**

S8. Fine-grained metric-scale diagnostics

Table S8 expands main-text Section 4.2.2 / Table 4 to per-sequence ρ, $\varepsilon s = |\rho - 1|$, translational / rotational RPE, and 50 m segment-wise drift. Mean ρ on usable clips is 90.17%; mean max $|\rho(k)-1|$ is 0.116. Prefix $\rho(t)$ versus frame index appears in Figure S1.

Table S8. Per-sequence metric-scale diagnostics for MoGe2-SLAM from saved paper-protocol trajectories.

Dataset	Sequence	ρ (%)	εs	RPEt (m)	RPEr (°)	std ρ(k)	max |ρ(k)−1|
KITTI	00	96.70	0.033	0.136	0.116	0.011	0.055
KITTI	02	93.03	0.070	0.206	0.115	0.009	0.083
KITTI	03	79.97	0.200	0.427	0.125	0.028	0.235
KITTI	05	97.81	0.022	0.146	0.120	0.036	0.073
KITTI	06	96.88	0.031	0.293	0.194	0.039	0.063
KITTI	07	98.79	0.012	0.086	0.142	0.023	0.038
KITTI	08	98.48	0.015	0.081	0.091	0.013	0.020
KITTI	09	95.29	0.047	0.167	0.134	0.028	0.058
KITTI	10	83.35	0.166	0.362	0.277	0.039	0.188

Waymo	100613	90.09	0.099	0.263	0.127	0.013	0.111
Waymo	106762	72.88	0.271	1.002	0.183	0.011	0.283
Waymo	132384	73.24	0.268	1.034	0.200	0.027	0.315
Waymo	13476	95.19	0.048	0.103	0.148	0.000	0.060
Waymo	152706	88.15	0.118	0.209	0.090	0.007	0.128
Waymo	153495	84.48	0.155	0.412	0.413	0.008	0.146
Waymo	158686	80.77	0.192	0.328	0.119	0.000	0.181
Waymo	163453	78.81	0.212	0.420	0.120	0.031	0.226
Waymo	405841	87.21	0.128	0.252	0.121	0.000	0.094
Argoverse 2	1ca5291b	92.9	0.071	2.054	1.947	0.000	0.071
Argoverse 2	2a930061	93.16	0.068	3.851	0.664	0.000	0.089
Argoverse 2	30e94a6b	94.14	0.059	2.576	0.604	0.000	0.059
Argoverse 2	3503b283	111.43	0.114	2.004	6.724	0.000	0.114
Argoverse 2	4612f4a4	92.02	0.080	2.603	1.076	0.000	0.094
Argoverse 2	48c9cd36	98.89	0.011	2.148	1.142	0.000	0.011
Argoverse 2	4fae2ef6	94.29	0.057	2.844	2.177	0.000	0.057
Argoverse 2	1ad57a00	94.74	0.053	2.844	2.177	0.000	0.053
Argoverse 2	14896a70	93.52	0.065	3.851	0.664	0.000	0.065

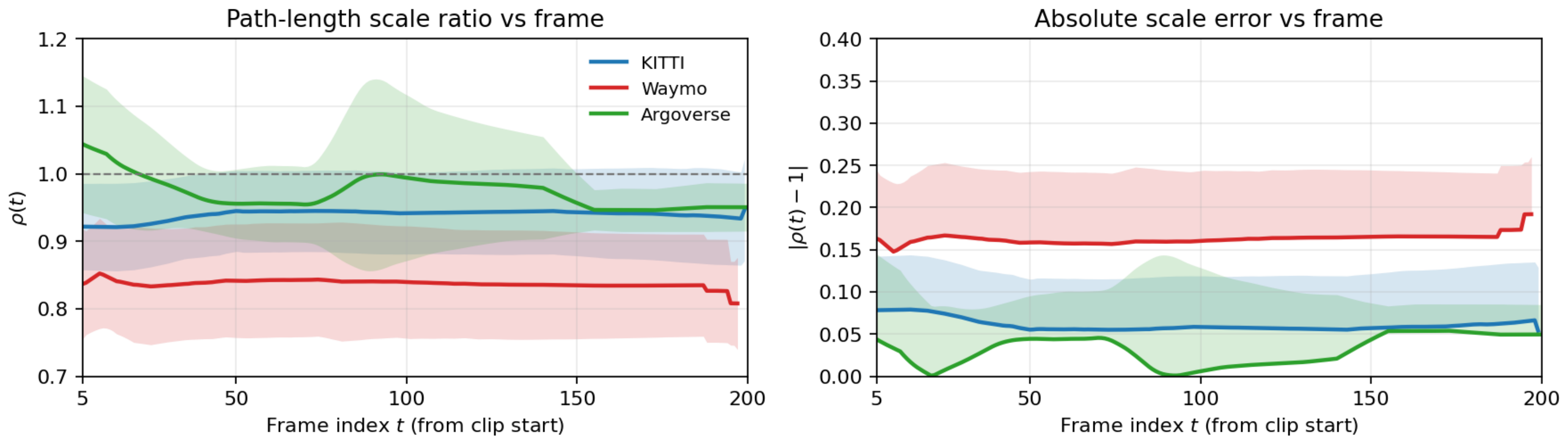

Figure S1. Scale error versus frame index (supports Section 4.2.2 / Table S7). Curves show mean ± std of the prefix path-length ratio ρ(t) (left) and |ρ(t)−1| (right) against frame index from the clip start. The dashed line marks ρ = 1. Trajectories are the same paper-protocol runs as Table S7.

S9–S13. Additional revision experiments

Table S9. Depth-prior swap on KITTI 00, 05, and 07 under identical Absolute-Scale Initialization, Grid-Balanced Tracking, and ORB-Anchored Mapping. MoGe-2 is the default prior used in the main text. Depth Pro / VGGT / Depth Anything2 replaces only the frozen dense depth / point-map; the camera-height ruler is unchanged.

Prior	00 Sim(3)	00 SE(3)	05 Sim(3)	05 SE(3)	07 Sim(3)	07 SE(3)	Mean Sim(3)	Mean SE(3)
**MoGe-2 (default)**	0.302	0.956	0.749	1.000	0.647	0.672	0.566	0.876
**Depth Pro**	0.351	1.94	0.957	1.88	0.491	1.97	0.600	1.93
**VGGT**	0.382	2.69	0.886	3.19	0.430	1.54	0.566	2.47
**Depth Anything2**	0.421	2.41	1.002	2.94	0.619	2.61	0.681	2.65

Table S10. Module ablation on nine Waymo FRONT clips. Row names match the main-text Table 8 convention: MASt3R w/ Camera height, MASt3R w/o Camera height, and MoGe-2 w/o Camera height.

Variant	ATE-Sim(3) ↓	ATE-SE(3) ↓	PSNR ↑	SSIM ↑	LPIPS ↓
MASt3R w/ Camera height	0.765	25.976	25.79	0.84	0.35
MASt3R w/o Camera height	0.731	27.174	26.11	0.87	0.34
MoGe-2 w/o Camera height	0.709	14.947	26.09	0.85	0.35
**w/o grid-balanced tracking**	0.936	6.889	25.98	0.83	0.36
**w/o ORB-anchored mapping**	0.716	7.183	26.02	0.82	0.39
**MoGe2-SLAM (full)**	0.693	5.463	26.68	0.85	0.31

Table S11. Module ablation on nine Argoverse 2 clips. Row names match the main-text Table 8 convention: MASt3R w/ Camera height, MASt3R w/o Camera height, and MoGe-2 w/o Camera height.

Variant	ATE-Sim(3) ↓	ATE-SE(3) ↓	PSNR ↑	SSIM ↑	LPIPS ↓
MASt3R w/ Camera height	0.596	20.673	25.73	0.77	0.42
MASt3R w/o Camera height	0.621	23.709	25.56	0.76	0.44
MoGe-2 w/o Camera height	0.639	9.337	25.44	0.76	0.44
**w/o grid-balanced tracking**	0.572	4.091	24.19	0.73	0.46
**w/o ORB-anchored mapping**	0.643	5.248	25.04	0.74	0.45
**MoGe2-SLAM (full)**	0.510	3.920	25.87	0.76	0.45

Table S12. Key hyperparameters used in all reported experiments unless a table caption states otherwise. Values are copied from the main text (Sections 3.2–3.4 and 4.1) for convenient lookup.

Parameter	Value	Main-text location
**Camera height h_c (KITTI)**	1.65 m	Section 4.1
**Camera height h_c (Waymo FRONT)**	2.12 m	Section 4.1
**Camera height h_c (Argoverse 2 ring_front_center)**	1.38 m	Section 4.1
**MoGe-2 checkpoint**	moge-2-vitl	Section 3.2.1
**Near-field ground ROI**	lower 40% of image height	Section 3.2.2
**Optional plane Tikhonov weight λ_n**	0.05 (0 if disabled)	Section 3.2.2
**Bootstrap RANSAC hypotheses M**	32	Section 3.2.3
**Optional h_c jitter**	±0.02 m	Section 3.2.3
**MAD rejection test**	MAD($\{s_0\}$) > 0.05 $\lvert s_0 \rvert$	Section 3.2.3

**Multi-plane fallback**	RMS / |d| > 0.10	Section 3.2.3
**Occupancy grid G**	8 (8×8)	Section 3.3.1
**Coverage weight η**	0.5	Section 3.3.1
**ORB nfeatures**	8000	Section 3.3.1
**Default FAST threshold τ_{FAST}**	5 (fallback 3, then 1)	Section 3.3.1
**Photometric pose polish**	Adam, 5 steps, 5×10^{-3}	Section 3.3 / Eq. (8)
**Per-frame tracking iteration budget**	up to 100	Section 3.3
**Mapping loss weight λ**	0.98	Section 3.4.3
**Isotropic regularizer λ_{iso}**	10	Section 3.4.3
**Sliding window |□|**	8	Section 3.4.3

Table S13. Prefix ATE-SE(3) and resource traces on extended KITTI logs. Columns 200/400/600 fr report keyframe ATE-SE(3) RMSE (m) at those lengths. Peak GPU, |G|, Trk, and Map are the same quantities as main-text Table 12: peak GPU memory (GB), active Gaussian count ($\times10^5$), mean per-frame tracking time (s), and mean keyframe mapping time (s). KITTI 07 is the long run that supports Figure 11; Peak GPU = 8.2 GB and |G| = 1.768×10^5 on that row match Table 12.

Sequence	200 fr	400 fr	600 fr	Peak GPU (GB)	|G| ($\times10^5$)	Trk (s)	Map (s)
**KITTI 07**	0.51	1.64	6.67	8.2	1.768	0.961	2.030
**KITTI 05**	0.95	2.35	3.87	8.8	1.842	0.879	1.838
**KITTI 03**	1.63	5.41	7.25	8.8	1.596	0.877	1.949
**KITTI 00**	0.30	1.43	4.89	8.6	1.893	0.977	2.142
**KITTI 06**	1.36	3.93	9.54	8.1	1.841	0.996	1.798
